# Supplementary material for: Effects of schistosomes on host anti-viral immune response and the acquisition, virulence, and prevention of viral infections: A systematic review
Source: PLoS Pathog. 2021 May 20;17(5):e1009555. doi: 10.1371/journal.ppat.1009555 (PMC8172021; doi:10.1371/journal.ppat.1009555)
Supplement: S1 Table — (DOCX) [file ppat.1009555.s001.docx]

## **S1 Table. List of Excluded Studies**

| **Title** | **Study** | **Reason for Exclusion** |
| --- | --- | --- |
| Acquired immunodeficiency syndrome (AIDS) and parasitic diseases in Puerto Rico. | Hillyer 1988 | Review piece/opinion piece/case series |
| Female genital schistosomiasis as a risk-factor for the transmission of HIV. | Feldmeier 1994 | Review piece/opinion piece/case series |
| Female genital schistosomiasis: a neglected risk factor for the transmission of HIV?. | Feldmeier 1995 | Review piece/opinion piece/case series |
| Hepatitis viruses, schistosomal infection and liver cancer in Egypt. | Bedwani 1996 | Review piece/opinion piece/case series |
| Schistosomiasis and associated infections. | Lambertucci 1998 | Review piece/opinion piece/case series |
| HIV infection and tropical parasitic diseases - deleterious interactions in both directions?. | Harms 2002 | Review piece/opinion piece/case series |
| Vaccination against helminths: Influence on HIV/AIDS and TB [1] | Fincham 2002 | Review piece/opinion piece/case series |
| Could control of soil-transmitted helminthic infection influence the HIV/AIDS pandemic. | Fincham 2003 | Review piece/opinion piece/case series |
| Symptomatic Schistosoma mansoni infection as an immune restoration phenomenon in a patient receiving antiretroviral therapy. | deSilva 2006 | Review piece/opinion piece/case series |
| Liver disease in Egypt: hepatitis C superseded schistosomiasis as a result of iatrogenic and biological factors. | Strickland 2006 | Review piece/opinion piece/case series |
| Interactions between schistosomiasis and infection with HIV-1. | Secor 2006 | Review piece/opinion piece/case series |
| Immune modulation by helminth infections | Helmby 2006 | Review piece/opinion piece/case series |
| Immune modulation by helminthic infections: Worms and viral infections | Kamal 2006 | Review piece/opinion piece/case series |
| Below the belt: new insights into potential complications of HIV-1/schistosome coinfections. | Secor 2007 | Review piece/opinion piece/case series |
| Treatment of helminth co-infection in individuals with HIV-1: A systematic review of the literature. | Walson 2007 | Review piece/opinion piece/case series |
| Treatment of helminth co-infection in HIV-1 infected individuals in resource-limited settings. | Walson 2008 | Review piece/opinion piece/case series |
| HIV/AIDS, schistosomiasis, and girls. | Stoever 2009 | Review piece/opinion piece/case series |
| Severe hepatosplenic schistosomiasis: clinicopathologic study of 102 cases undergoing splenectomy. | Li 2011 | Review piece/opinion piece/case series |
| HBV and HCV serological markers in patients with the hepatosplenic form of mansonic schistosomiasis. | Silva 2011 | Review piece/opinion piece/case series |
| Effect of schistosoma infection on malaria immune response: A systematic review. | Yesuf 2011 | Review piece/opinion piece/case series |
| Species-specific treatment effects of helminth/HIV-1 co-infection: a systematic review and meta-analysis. | Sangare 2011 | Review piece/opinion piece/case series |
| Examining the relationship between urogenital schistosomiasis and HIV infection. | Mbabazi 2011 | Review piece/opinion piece/case series |
| Effects of helminths and Mycobacterium tuberculosis infection on HIV-1: A cellular immunological perspective | Mouser 2012 | Review piece/opinion piece/case series |
| The effects of schistosomiasis on HIV/AIDS infection, progression and transmission. | Secor 2012 | Review piece/opinion piece/case series |
| Helminth infections and host immune regulation | McSorley 2012 | Review piece/opinion piece/case series |
| Cervical squamous intraepithelial lesions and associated cervical infections in an HIV-positive population in Rural Mpumalanga, South Africa. | Swanepoel 2013 | Review piece/opinion piece/case series |
| Study on *Schistosomiasis mansoni* and Comborbidity with Hepatitis B and C Virus Infection | Conceiçāo 2018 | Review piece/opinion piece/case series |
| Association between Schistosomiasis mansoni and hepatitis C: systematic review | Van-Lume 2013 | Review piece/opinion piece/case series |
| Neglected tropical diseases as a cause of chronic liver disease: the case of Schistosomiasis and Hepatitis C Co-infections in Egypt. | Sanghvi 2013 | Review piece/opinion piece/case series |
| Epidemiology and interactions of Human Immunodeficiency Virus - 1 and Schistosoma mansoni in sub-Saharan Africa. | Mazigo 2013 | Review piece/opinion piece/case series |
| Examining the relationship between urogenital schistosomiasis and HIV infection | Mbabazi 2013 | Review piece/opinion piece/case series |
| A helminth-mediated viral awakening. | Veldhoen 2014 | Review piece/opinion piece/case series |
| Genital schistosomiasis and its unacknowledged role on HIV transmission in the STD intervention studies | Kjetland 2014 | Review piece/opinion piece/case series |
| HIV and schistosomiasis co-infection in African children | Bustinduy 2014 | Review piece/opinion piece/case series |
| Are pregnant women with chronic helminth infections more susceptible to congenital infections? | Abdoli 2014 | Review piece/opinion piece/case series |
| Immune system modulation by helminth infections: Potential impact on HIV transmission and disease progression | Chachage 2014 | Review piece/opinion piece/case series |
| Interaction Between the Neglected Tropical Disease Human Schistosomiasis and HCV Infection in Egypt: a Puzzling Relationship. | Bahgat 2014 | Review piece/opinion piece/case series |
| Schistosomiasis, hepatitis B and hepatitis C co-infection. | Gasim 2015 | Review piece/opinion piece/case series |
| Considering treatment of male genital schistosomiasis as a tool for future HIV prevention: a systematic review. | Stecher 2015 | Review piece/opinion piece/case series |
| Female genital schistosomiasis and human immunodeficiency virus infection: A systematic literature review | Christinet 2015 | Review piece/opinion piece/case series |
| Antihelminthics in helminth-endemic areas: effects on HIV disease progression. | Means 2016 | Review piece/opinion piece/case series |
| Antihelminthics in helminth-endemic areas: Effects on HIV disease progression | Means 2016 | Review piece/opinion piece/case series |
| A Novel Mechanism of Immune Memory Unveiled at the Invertebrate-Parasite Interface | Coustau 2016 | Review piece/opinion piece/case series |
| Coinfection of Schistosoma Species with Hepatitis B or Hepatitis C Viruses. | Abruzzi 2016 | Review piece/opinion piece/case series |
| Prevalence of intestinal parasites in newly diagnosed HIV/AIDS patients in Ilorin, Nigeria | Obateru 2017 | Review piece/opinion piece/case series* |
| A Systematic Review of the Risk of HIV Transmission with Concurrent Schistosomiasis Infection | Lee 2018 | Review piece/opinion piece/case series |
| Schistosomiasis and hepatitis B infection in pregnancy: implications for vaccination against hepatitis B. | Patana 1995 | Outcomes not related to control or prevention of virus |
| Chronic hepatitis in experimental schistosomiasis. | Andrade 1995 | Outcomes not related to control or prevention of virus |
| Reversibility of lower reproductive tract abnormalities in women with Schistosoma haematobium infection after treatment with praziquantel--an interim report. | Richter 1996 | Outcomes not related to control or prevention of virus |
| Impact of hepatitis C virus infection on schistosomal liver disease. | Mohamed 1998 | Outcomes not related to control or prevention of virus |
| Risk factors for hepatocellular carcinoma in Egypt: the role of hepatitis-B viral infection and schistosomiasis. | Badawi 1999 | Outcomes not related to control or prevention of virus |
| Inhibition of hepatitis B virus replication during schistosoma mansoni infection in transgenic mice. | McClary 2000 | Outcomes not related to control or prevention of virus |
| Prevalence of hepatitis C virus (HCV) infection and its vertical transmission in Egyptian pregnant women and their newborns. | Kassem 2000 | Outcomes not related to control or prevention of virus |
| Female genital schistosomiasis of the lower genital tract: prevalence and disease-associated morbidity in northern Tanzania. | Poggensee 2000 | Outcomes not related to control or prevention of virus |
| Schistosomiasis mansoni and viral B hepatitis in woodchucks. | Andrade 2001 | Outcomes not related to control or prevention of virus |
| Further evidence for association of hepatitis C infection with parenteral schistosomiasis treatment in Egypt. | Rao 2002 | Outcomes not related to control or prevention of virus |
| Generation of Th1 immune responses to inactivated, gp120-depleted HIV-1 in mice with a dominant Th2 biased immune profile via imunostimulatory oligonucleotides - Relevance to AIDS vaccines in developing countries | Ayash-Rashkovsky 2002 | Outcomes not related to control or prevention of virus |
| Interactions between Schistosoma haematobium and human immunodeficiency virus type 1: the effects of coinfection on treatment outcomes in rural Zambia. | Mwanakasale 2003 | Outcomes not related to control or prevention of virus |
| Non-invasive markers and predictors of severity of hepatic fibrosis in HCV patients at Sharkia Governorate, Egypt. | el-Shorbagy 2004 | Outcomes not related to control or prevention of virus |
| Impairment of the Schistosoma mansoni-specific immune responses elicited by treatment with praziquantel in Ugandans with HIV-1 coinfection. | Joseph 2004 | Outcomes not related to control or prevention of virus |
| Sensitive detection of human papillomavirus in cervical, head/neck, and schistosomiasis-associated bladder malignancies. | Yang 2005 | Outcomes not related to control or prevention of virus |
| Schistosomiasis and HIV-1 infection in rural Zimbabwe: implications of coinfection for excretion of eggs. | Kallestrup 2005 | Outcomes not related to control or prevention of virus |
| Possible contribution of serum activin A and IGF-1 in the development of hepatocellular carcinoma in Egyptian patients suffering from combined hepatitis C virus infection and hepatic schistosomiasis. | Elsammak 2006 | Outcomes not related to control or prevention of virus |
| Schistosomiasis and HIV in rural Zimbabwe: efficacy of treatment of schistosomiasis in individuals with HIV coinfection. | Kallestrup 2006 | Outcomes not related to control or prevention of virus |
| Assessment of platelet activation in coupled schistosomiasis and viral hepatitis infection: contribution to complexity of course and development of complications. | Hussein 2006 | Outcomes not related to control or prevention of virus |
| Cytokines and immunoglobulin-E in certain parasitic diseases. | Elshazly 2006 | Outcomes not related to control or prevention of virus |
| Reduced mortality and CD4 cell loss among carriers of the interleukin-10 -1082G allele in a Zimbabwean cohort of HIV-1-infected adults. | Erikstrup 2007 | Outcomes not related to control or prevention of virus |
| Seroprevalence of schistosomiasis in African patients infected with HIV. | Smith 2008 | Outcomes not related to control or prevention of virus |
| Downregulation of MIP-1alpha/CCL3 with praziquantel treatment in Schistosoma haematobium and HIV-1 co-infected individuals in a rural community in Zimbabwe. | Zinyama-Gutsire 2009 | Outcomes not related to control or prevention of virus |
| IgE-FcepsilonRI interactions determine HIV coreceptor usage and susceptibility to infection during ontogeny of mast cells. | Sundstrom 2009 | Outcomes not related to control or prevention of virus |
| Influence of maternal schistosomiasis on the immunity of adult offspring mice | Santos 2010 | Outcomes not related to control or prevention of virus |
| Helminth antigens as adjuvants for HIV-1 vaccines | Bui 2011 | Outcomes not related to control or prevention of virus |
| Increased levels of HIV target cells and vascularity in female genital mucosa with Schistosoma haematobium infection | Jourdan 2011 | Outcomes not related to control or prevention of virus |
| HIV-1 vaccine-specific responses induced by Listeria vector vaccines are maintained in mice subsequently infected with a model helminth parasite, Schistosoma mansoni. | Shollenberger 2013 | Outcomes not related to control or prevention of virus |
| CD14 influences host immune responses and alternative activation of macrophages during Schistosoma mansoni infection | Tundup 2014 | Outcomes not related to control or prevention of virus |
| Schistosoma mansoni soluble egg antigens enhance Listeria monocytogenes vector HIV-1 vaccine induction of cytotoxic T cells. | Bui 2014 | Outcomes not related to control or prevention of virus |
| Schistosoma mansoni soluble egg antigens enhance T cell responses to a newly identified HIV-1 Gag H-2b epitope. | Bui 2015 | Outcomes not related to control or prevention of virus |
| HIV and schistosomiasis in rural Zimbabwe: the association of retinol-binding protein with disease progression, inflammation and mortality. | Kotze 2015 | Outcomes not related to control or prevention of virus |
| Periportal fibrosis, liver and spleen sizes among S. mansoni mono or co-infected individuals with human immunodeficiency virus-1 in fishing villages along Lake Victoria shores, North-Western, Tanzania. | Mazigo 2015 | Outcomes not related to control or prevention of virus |
| A Phase Ib Study of the Safety, Reactogenicity, and Immunogenicity of Sm-TSP-2/Alhydrogel)(R) With or Without AP 10-701 for Intestinal Schistosomiasis in Healthy Exposed Adults | ID - NCT03110757 | Outcomes not related to control or prevention of virus |
| Sensitivity and specificity of point-of-care circulating Cathodic antigen test before and after praziquantel treatment in diagnosing Schistosoma mansoni infection in adult population co-infected with human immunodeficiency virus-1, North-Western Tanzania. | Mazigo 2018 | Outcomes not related to control or prevention of virus |
| Decreased Sensitivity of Schistosoma sp. Egg Microscopy in Women and HIV-Infected Individuals. | Colombe 2018 | Outcomes not related to control or prevention of virus |
| Mother-to-child transmission of hepatitis B virus in a semirural population in Egypt. | Ghaffar 1989 | CAA testing or egg excretion not used for diagnosis of schistosomiasis |
| Safety and immunogenicity of a recombinant hepatitis B vaccine in patients infected with Schistosoma mansoni. | Bassily 1990 | CAA testing or egg excretion not used for diagnosis of schistosomiasis |
| Hepatitis B virus infection among immunocompromised patients in Egypt. | Darwish 1990 | CAA testing or egg excretion not used for diagnosis of schistosomiasis |
| Hepatitis-B virus and schistosomiasis infections in childhood proteinuria. | Zeid 1994 | CAA testing or egg excretion not used for diagnosis of schistosomiasis |
| Does schistosomiasis play a role in the high sero prevalence of HCV antibody among Egyptians?. | el-Zayadi 1997 | CAA testing or egg excretion not used for diagnosis of schistosomiasis |
| The relationship between hepatitis C virus and schistosomiasis: histopathologic evaluation of liver biopsy specimens. | Helal 1998 | CAA testing or egg excretion not used for diagnosis of schistosomiasis |
| High prevalence of GB-C/hepatitis G virus in a Brazilian population with helminth infection. | Gallian 1998 | CAA testing or egg excretion not used for diagnosis of schistosomiasis |
| Evaluation of nitric oxide (NO) levels in hepatitis C virus (HCV) infection: relationship to schistosomiasis and liver cirrhosis among Egyptian patients. | Hassan 2002 | CAA testing or egg excretion not used for diagnosis of schistosomiasis |
| Interactions between schistosomiasis and human immunodeficiency virus in Western Kenya. | Secor 2004 | CAA testing or egg excretion not used for diagnosis of schistosomiasis |
| HCV and associated concomitant infections at Sharkia Governorate, Egypt. | Mangoud 2004 | CAA testing or egg excretion not used for diagnosis of schistosomiasis |
| Schistosoma infection inhibits cellular immune responses to core HCV peptides. | Farid 2005 | CAA testing or egg excretion not used for diagnosis of schistosomiasis |
| A cross sectional study of hepatitis B, C, some trace elements, heavy metals, aflatoxin B1 and schistosomiasis in a rural population, Egypt. | Sayed 2005 | CAA testing or egg excretion not used for diagnosis of schistosomiasis |
| INF-gamma, IL-5 and IgE profiles in chronic schistosomiasis mansoni Egyptian patients with or without hepatitis C infection. | Makhlouf 2006 | CAA testing or egg excretion not used for diagnosis of schistosomiasis |
| Seroprevalence and risk factors for human herpesvirus 8 infection, rural Egypt. | Mbulaiteye 2008 | CAA testing or egg excretion not used for diagnosis of schistosomiasis |
| Schistosoma mansoni coinfection could have a protective effect against mixed cryoglobulinaemia in hepatitis C patients. | Abbas 2009 | CAA testing or egg excretion not used for diagnosis of schistosomiasis |
| Modeling schistosomiasis and HIV/AIDS codynamics. | Mushayabasa 2011 | CAA testing or egg excretion not used for diagnosis of schistosomiasis |
| IL-4 and IFN-gamma induced by human immunodeficiency virus vaccine in a schistosome infection model. | Yin 2012 | CAA testing or egg excretion not used for diagnosis of schistosomiasis |
| HIV and Schistosoma haematobium prevalences correlate in sub-Saharan Africa. | Mbah 2013 | CAA testing or egg excretion not used for diagnosis of schistosomiasis |
| Coinfection with hepatitis C virus and schistosomiasis: fibrosis and treatment response. | Abdel-Rahman 2013 | CAA testing or egg excretion not used for diagnosis of schistosomiasis |
| Schistosomiasis does not affect the outcome of HCV infection in genotype 4-infected patients. | Allam 2014 | CAA testing or egg excretion not used for diagnosis of schistosomiasis |
| A cross-sectional serological study of cysticercosis, schistosomiasis, toxocariasis and echinococcosis in HIV-1 infected people in Beira, Mozambique. | Noormahomed 2014 | CAA testing or egg excretion not used for diagnosis of schistosomiasis |
| P selectins and immunological profiles in HCV and Schistosoma mansoni induced chronic liver disease. | Kamel 2014 | CAA testing or egg excretion not used for diagnosis of schistosomiasis |
| Transcriptional profiling of chronic clinical hepatic schistosomiasis japonica indicates reduced metabolism and immune responses | Gobert 2015 | CAA testing or egg excretion not used for diagnosis of schistosomiasis |
| AN INITIAL INDICATION OF PREDISPOSING RISK OF SCHISTOSOMA MANSONI INFECTION FOR HEPATOCELLULAR CARCINOMA | SabryAe 2015 | CAA testing or egg excretion not used for diagnosis of schistosomiasis |
| Association Between Schistosoma haematobium Exposure and Human Immunodeficiency Virus Infection Among Females in Mozambique. | Brodish 2016 | CAA testing or egg excretion not used for diagnosis of schistosomiasis |
| Correlation of Interferon-gamma and Interleukin-28B levels in patients with chronic hepatitis C viral infection with or without Schistosoma mansoni coinfection | Eid 2016 | CAA testing or egg excretion not used for diagnosis of schistosomiasis |
| HCV Infection Amplified Th2 Bias and Th17 Responses In Schistosoma-Infected Patients. | Nady 2016 | CAA testing or egg excretion not used for diagnosis of schistosomiasis |
| Impact of schistosomiasis on increase incidence of occult hepatitis B in chronic hepatitis C patients in Egypt | Taha 2017 | CAA testing or egg excretion not used for diagnosis of schistosomiasis |
| Schistosomiasis is associated with incident HIV transmission and death in Zambia. | Wall 2018 | CAA testing or egg excretion not used for diagnosis of schistosomiasis |
| Immune Dysfunction and Coinfection with Human Immunodeficiency Virus and Schistosoma japonicum in Yi People. | Yang 2018 | CAA testing or egg excretion not used for diagnosis of schistosomiasis |
| Coinfections between persistent parasitic neglected tropical diseases and viral infections among prisoners from Sub-Saharan Africa and Latin America | DaSilvaSantos 2018 | CAA testing or egg excretion not used for diagnosis of schistosomiasis |
| Integrated Cross-Sectional Multiplex Serosurveillance of IgG Antibody Responses to Parasitic Diseases and Vaccines in Coastal Kenya. | Njenga 2019 | CAA testing or egg excretion not used for diagnosis of schistosomiasis |
| A case-control study on liver cancer with special emphasis on the possible aetiological role of schistosomiasis. | Inaba 1984 | Examined coinfection with non-viruses |
| Immunological response to diphtheria/tetanus vaccine in Schistosomiasis mansoni patients. | elGhorab 1992 | Examined coinfection with non-viruses |
| Nontyphoidal salmonellal septicemia in Gabonese children infected with Schistosoma intercalatum. | Gendrel 1994 | Examined coinfection with non-viruses |
| Impairment of tetanus toxoid-specific Th1-like immune responses in humans infected with Schistosoma mansoni. | Sabin 1996 | Examined coinfection with non-viruses |
| Antibody response to Salmonella typhi in human schistosomiasis mansoni. | Muniz-Junqueira 1996 | Examined coinfection with non-viruses |
| Altered immune responses in mice with concomitant Schistosoma mansoni and Plasmodium chabaudi infections. | Helmby 1998 | Examined coinfection with non-viruses |
| Schistosoma mansoni infection reduces the protective efficacy of BCG vaccination against virulent Mycobacterium tuberculosis. | Elias 2005 | Examined coinfection with non-viruses |
| [Interaction between malaria and intestinal helminthiasis in Senegal: influence of the carriage of intestinal parasites on the intensity of the malaria infection]. | Faye 2008 | Examined coinfection with non-viruses |
| Reduced protective effect of Plasmodium berghei immunization by concurrent Schistosoma mansoni infection. | Laranjeiras 2008 | Examined coinfection with non-viruses |
| Plasmodium falciparum and helminth coinfection in a semi urban population of pregnant women in Uganda. | Hillier 2008 | Examined coinfection with non-viruses |
| Schistosomiasis coinfection in children influences acquired immune response against Plasmodium falciparum malaria antigens. | Diallo 2010 | Examined coinfection with non-viruses |
| Associations between peripheral Plasmodium falciparum malaria parasitemia, human immunodeficiency virus, and concurrent helminthic infection among pregnant women in Malawi. | Thigpen 2011 | Examined coinfection with non-viruses |
| Pre-existing Schistosoma japonicum infection alters the immune response to Plasmodium berghei infection in C57BL/6 mice. | Wang 2013 | Examined coinfection with non-viruses |
| Associations between maternal helminth and malaria infections in pregnancy and clinical malaria in the offspring: a birth cohort in entebbe, Uganda. | Ndibazza 2013 | Examined coinfection with non-viruses |
| Successful vaccination of immune suppressed recipients using Listeria vector HIV-1 vaccines in helminth infected mice. | Shollenberger 2013 | Examined coinfection with non-viruses |
| The effect of maternal helminth infection on maternal and neonatal immune function and immunity to tuberculosis. | Gebreegziabiher 2014 | Examined coinfection with non-viruses |
| Helminth-M. tb Co-Infection | duPlessis 2014 | Examined coinfection with non-viruses |
| Impact of Schistosoma mansoni on malaria transmission in Sub-Saharan Africa. | NdeffoMbah 2014 | Examined coinfection with non-viruses |
| Coinfection with Plasmodium falciparum and Schistosoma haematobium: additional evidence of the protective effect of Schistosomiasis on malaria in Senegalese children. | Lemaitre 2014 | Examined coinfection with non-viruses |
| Low to moderate intensity schistosoma mansoni infections do not alter protective antibody responses to tetanus toxoid booster immunizations | Riner 2015 | Examined coinfection with non-viruses |
| Maternal parasitic infections during pregnancy and specific anti-parasite cytokine responses in cord blood are associated with impaired vaccine efficacy in Kenyan infants | Malhotra 2015 | Examined coinfection with non-viruses |
| Maternal parasitic infections alter infant antibody response to pneumococcal immunization | McKittrick 2016 | Examined coinfection with non-viruses |
| Effect of prenatal exposure to schistosomiasis and co-infections with schistosomiasis on fetal immune responses | Nyakundi 2016 | Examined coinfection with non-viruses |
| Mycobacterium tuberculosis-specific CD4+ T-cell response is increased, and Treg cells decreased, in anthelmintic-treated patients with latent TB. | Toulza 2016 | Examined coinfection with non-viruses |
| Anti-Schistosoma IgG responses in Schistosoma haematobium single and concomitant infection with malaria parasites. | Morenikeji 2016 | Examined coinfection with non-viruses |
| Maternal schistosoma mansoni infection alters the immune response of offspring to tetanus and diphteria immunization | Selva 2016 | Examined coinfection with non-viruses |
| Altered fetal immune responses by prenatal exposure to maternal co-infections | Nyakundi 2017 | Examined coinfection with non-viruses |
| The effect of current Schistosoma mansoni infection on the immunogenicity of a candidate TB vaccine, MVA85A, in BCG-vaccinated adolescents: An open-label trial. | Wajja 2017 | Examined coinfection with non-viruses |
| The impact of IL4 serum concentration on the seroprevalence of schistosomiasis and chlamydia among HIV patients in Limpopo Province, South Africa | Mafokwane 2018 | Examined coinfection with non-viruses |
| Parasitic infections during pregnancy need not affect infant antibody responses to early vaccination against streptococcus pneumoniae, diphtheria, or haemophilus influenzae type B | McKittrick 2019 | Examined coinfection with non-viruses |
| Antagonistic effects of Plasmodium-helminth co-infections on malaria pathology in different population groups in Cote d'Ivoire. | Hurlimann 2019 | Examined coinfection with non-viruses |
| Mouse hepatitis virus (MHV3) infection in chronic murine schistosomiasis mansoni. | Warren 1969 | Included in Abruzzi review |
| Behaviour of hepatitis B antigen in bilharzial patients infected with HBs positive viral hepatitis. | Nooman 1978 | Included in Abruzzi review |
| Preliminary investigation on serum markers of hepatitis B virus in patients with schistosomiasis japonica. | Cai 1985 | Included in Abruzzi review |
| Hepatitis B virus vs schistosomiasis and hepatocellular carcinoma in Saudi Arabia. | Nouh 1990 | Included in Abruzzi review |
| Lack of association between schistosomiasis and hepatitis B virus infection in Gezira-Managil area, Sudan. | Eltoum 1991 | Included in Abruzzi review |
| Hepatitis B & D viral infections among schistosomal patients in Egypt. | Darwish 1992 | Included in Abruzzi review |
| Antibody to hepatitis C virus in patients with chronic schistosomiasis. | Uemura 1992 | Included in Abruzzi review |
| The effect of praziquantel administration on the course of hepatitis B among cases with concomitant schistosome infection. | Farghaly 1993 | Included in Abruzzi review |
| Delta virus versus HBsAg in chronic active hepatitis and their relation to clinical, laboratory, and morbidity findings in bilharzial and non-bilharzial patients. | el-Hawey 1993 | Included in Abruzzi review |
| The epidemiology of Schistosoma mansoni, hepatitis B and hepatitis C infection in Egypt. | Kamel 1994 | Included in Abruzzi review |
| Prevalence of hepatitis-C antibody seropositivity in healthy Egyptian children and four high risk groups. | el-Nanawy 1995 | Included in Abruzzi review |
| Urinary schistosomiasis associated with hepatitis C virus infection. | Koshy 1995 | Included in Abruzzi review |
| Risk of hepatitis "E" virus infection among some schistosomiasis patients in Egypt. | AbdelRahman 1995 | Included in Abruzzi review |
| Study of the high prevalence of HCV in Egypt. | Darwish 1995 | Included in Abruzzi review |
| Hepatitis C virus infection in Schistosomiasis mansoni in Brazil. | Pereira 1995 | Included in Abruzzi review |
| Absence of relationship between Schistosoma japonicum and hepatitis B virus infection in the Dongting lake region, China. | Ye 1998 | Included in Abruzzi review |
| Clinical, virological and histopathological features: long-term follow-up in patients with chronic hepatitis C co-infected with S. mansoni. | Kamal 2000 | Included in Abruzzi review |
| Biochemical changes in patients with combined chronic schistosomiasis and viral hepatitis C infections. | Fahim 2000 | Included in Abruzzi review |
| Relationship between hepatitis C virus infection and schistosomal liver disease: not simply an additive effect. | Gad 2001 | Included in Abruzzi review |
| Prevalence of hepatitis C virus among bilharziasis patients. | Khan 2004 | Included in Abruzzi review |
| Very low prevalence of hepatitis C virus infection in rural communities of northeastern Brazil with a high prevalence of schistosomiasis mansoni. | Tavares-Neto 2005 | Included in Abruzzi review |
| Progression of fibrosis in hepatitis C with and without schistosomiasis: correlation with serum markers of fibrosis. | Kamal 2006 | Included in Abruzzi review |
| Intensity of Schistosoma mansoni, hepatitis B, age, and sex predict levels of hepatic periportal thickening/fibrosis (PPT/F): a large-scale community-based study in Ethiopia. | Berhe 2007 | Included in Abruzzi review |
| Chronic hepatitis B and liver schistosomiasis: a deleterious association. | Andrade 2014 | Included in Abruzzi review |
| Schistosome infection aggravates HCV-related liver disease and induces changes in the regulatory T-cell phenotype. | Loffredo-Verde 2015 | Included in Abruzzi review |
| Co-infection of Schistosoma mansoni/hepatitis C virus and their associated factors among adult individuals living in fishing villages, north-western Tanzania. | Mazigo 2017 | Included in Abruzzi review |
| Generation of Th1 immune responses to inactivated, gp120-depleted HIV-1 in mice with a dominant Th2 biased immune profile via immunostimulatory [correction of imunostimulatory] oligonucleotides--relevance to AIDS vaccines in developing countries. | Ayash-Rashkovsky 2002 | Duplicate |
| Different cytokine patterns in patients coinfected with hepatitis C virus and Schistosoma mansoni. | el-Kady 2004 | Duplicate |
| Antenatal malaria and helminth infections are associated with impaired vaccine efficacy in Kenyan infants | Malhotra 2011 | Duplicate |
| Antenatal malaria infections are associated with impaired hib and diphtheria vaccine immune responses in kenyan children | Malhotra 2012 | Duplicate |
| Schistosomiasis during pregnancy is associated with impaired vaccine efficacy in Kenyan infants | Malhotra 2013 | Duplicate |
| Impact of schistosomiasis and soil transmitted helminths on vaccine-induced immune responses | Riner 2014 | Duplicate |
| Helminth infections during pregnancy is associated with impaired vaccine efficacy in Kenyan infants | Malhotra 2014 | Duplicate |
| Chronic helminthiasis suppresses both T Cell and antibody responses to DNA-MVA and MVA-protein HIV vaccine regimens despite anti-helminthic treatment | Dzhivhuho 2016 | Duplicate |
| Chronic helminthiasis prevents optimal boosting of T Cell and antibody responses by an MVA-Env protein HIV vaccine regimen in a mouse model | Humby 2016 | Duplicate |
| Cord blood anti-parasite IL-10 as risk marker for compromised vaccine immunogenicity in early childhood | Malhotra 2017 | Duplicate |
| Helminth-induced immunomodulation enhances rather than suppresses anti-viral immunity via IFN-gamma and Granzyme B in a dynamic pattern | LoffredoVerde 2017 | Duplicate |
| Treatment of schistosoma mansoni infection in ugandan women reduces HIV entry into cervical CD4 T cells and induces type i interferon pathways | Yegorov 2018 | Duplicate |
| Schistosoma mansoni eggs in the absence of live worms suppresses induction of HIV-1 envspecific antibody responses | Dzhivhuho 2018 | Duplicate |
| Immunogenicity of hepatitis B vaccine in patients infected with Schistosoma mansoni. | Bassily 1987 | Population all schisto infected |
| Hepatitis B vaccination in patients infected with Schistosoma mansoni: duration of immunity and immunogenicity of a low dose intradermal booster. | Bassily 1990 | Population all schisto infected |
| Hepatitis C virus antibodies in parasitic infections. | Aceti 1990 | Population all schisto infected |
| Specific liver autoreactivity in schistosomiasis mansoni. | Pereira 1997 | Population all schisto infected |
| Hepatitis B and C virus markers among patients with hepatosplenic mansonic schistosomiasis. | Aquino 2000 | Population all schisto infected |
| Observation of T lymphocyte subsets in the liver of patients with advanced schistosomiasis and advanced schistosomiasis accompanied with hepatitis B. | Zhang 2000 | Population all schisto infected |
| Cellular immune responses of schistosomiasis patients are altered by human immunodeficiency virus type 1 coinfection. | Mwinzi 2001 | Population all schisto infected |
| Generation of Th1 immune responses to inactivated, gp120-depleted HIV-1 in mice with a dominant Th2 biased immune profile via imunostimulatory oligonucleotides - Relevance to AIDS vaccines in developing countries | Ayash-Rashkovsky 2002 | Population all schisto infected |
| Short report: Evaluation of hepatic fibrosis in persons co-infected with Schistosoma mansoni and human immunodeficiency virus 1. | Mwinzi 2004 | Population all schisto infected |
| HTLV-1 modifies the clinical and immunological response to schistosomiasis. | Porto 2004 | Population all schisto infected |
| Hepatitis C and B virus in schistosomiasis patients on oral or parenteral treatment. | El-Sabah 2011 | Population all schisto infected |
| Hepatitis E virus seroprevalence among schistosomiasis patients in Northeastern Brazil. | Passos-Castilho 2016 | Population all schisto infected |
| [Hepatitides in infectious diseases]. | Teichmann 1972 | Not available in English |
| [E antigen (HBeAG) and surface antigen (HBsAg) in bladder schistosomiasis]. | Nuti 1978 | Not available in English |
| [Hepatitis B antigens systems in schistosomiasis mansoni]. | Guimaraes 1981 | Not available in English |
| [Humoral immune response to typhoid vaccine in hepatosplenic schistosomiasis mansoni]. | ShikanaiYasuda 1982 | Not available in English |
| [Bilharziasis and human immunodeficiency virus infection in Congo]. | N'Zoukoudi-N'Doundou 1995 | Not available in English |
| [Impact of chronic schistosomiasis japonica on the protective immunity induced by vaccine against hepatitis B virus]. | Song 2005 | Not available in English |
| [Schistosomiasis--a probable risk of HIV transmission] | Holen 2012 | Not available in English |
| Relationship between advanced schistosomiasis and HBV infection | Du 2013 | Not available in English |
| Co-infection malaria-helminthiasis in pregnant women at the general hospital of Kimpese, Democratic Republic of Congo | Umesumbu 2017 | Not available in English |
| The genital Schistosomiasis and HIV research project (GENSHIV): Effect of praziquantel treatment on genital HIV-1 RNA shedding in Schistosoma haematobium and HIV co-infected subjects-a randomized trial | Stecher 2013 | Presents a study protocol, not results |
| Safety and Immunogenicity Study of a DNA Vaccine Combined With Protein Vaccine Against HIV/AIDS | NCT02376582 2014 | Presents a study protocol, not results |
| The effect of intensive treatment for schistosomiasis on response to vaccines among island adolescents in Uganda | ISRCTN60517191 2019 | Presents a study protocol, not results |
| Immunoglobulins in bilharzial patients with and without chronic hepatitis. | Hamadto 1989 | Unable to be obtained |
| The role of intrahepaticT-Cell in immunopathogenesis of the liver cirrhosis in chronic hepatitis C with and without Schistosomiasis | Abdelaal 2013 | Abstract without enough information |
