# Supplementary material for: Effects of schistosomes on host anti-viral immune response and the acquisition, virulence, and prevention of viral infections: A systematic review
Source: PLoS Pathog. 2021 May 20;17(5):e1009555. doi: 10.1371/journal.ppat.1009555 (PMC8172021; doi:10.1371/journal.ppat.1009555)
Supplement: S2 Table — (DOCX) [file ppat.1009555.s002.docx]

## **S2 Table. Characteristics, Key Findings, and Quality Assessment of Included Studies.**

| **Virus** | **First author, year** | **Study type** | **Country** | **Study population** | **Schistosoma Species** | **Sample Size** | **Key Findings** | **Limitations /potential sources of bias** |
| --- | --- | --- | --- | --- | --- | --- | --- | --- |
| Hepatitis C virus | **Kamal 2001 (Gastroenter-ology) [20]** | Prospective cohort | Egypt | Human, Individuals with acute HCV with and without schistosome infection | *S. mansoni* | 32 | Coinfected individuals had lower HCV-specific CD4+ T-cell responses than HCV-monoinfected individuals. Coinfected individuals also developed a Th2-dominant response whereas HCV-monoinfected individuals developed a Th1-dominant response. | The study described CD4+ responses in peripheral blood but not in the liver where tissue damage occurs. |
| Hepatitis C virus | **Kamal 2001 (J Infect Dis) [21]** | Prospective cohort | Egypt | Human, Individuals with HCV alone; individuals with *S. mansoni* alone; individuals with HCV-*S. mansoni* coinfection; healthy matched controls | *S. mansoni* | 85 | Schistosome-HCV-coinfected individuals had decreased HCV-specific CD4+ T-cell responses compared to those with HCV alone. Coinfected individuals also had elevated IL-4 and IL-10. | In this study, the temporal order of HCV and schistosome infection acquisition is assumed, but not known. Additionally, this study did not assess cytotoxic responses. |
| Hepatitis C virus | **Kamel 2014 [22]** | Cross-sectional | Egypt | Human, Individuals with HCV alone; individuals with *S. mansoni* alone; individuals with HCV-*S. mansoni* coinfection; healthy controls | *S. mansoni* | 82 | No significant differences in percentage of peripheral blood CD4+ or CD8+ T cells between those with schistosome alone versus those with schistosome-HCV coinfection. | Small cross-sectional study. No discussion of how controls were selected. |
| Hepatitis C virus | **Loffredo-Verde 2015 [23]** | Cross-sectional | Egypt | Human, Individuals with HCV alone; individuals with HCV-*S. mansoni* coinfection; healthy matched controls | *S. mansoni* | 44 | Individuals coinfected with HCV and *S. mansoni* had similar CD4+ and CD8+ T cell frequencies as those with HCV alone, but higher granzymeB+ T regulatory cell frequencies. Coinfected individuals had higher lower IL-6 and higher HCV RNA viral loads. | This study had a small sample size and did not control for multiple statistical comparisons. |
| Hepatitis C virus | **Elrefaei 2003 [24]** | Cross-sectional | Egypt | Human, Individuals with chronic HCV infection with and without schistosome infection | *S. mansoni* | 24 | HCV-schistosome coinfected individuals had fewer late differentiated HCV-specific memory T cells than HCV monoinfected individuals. | This study had a small sample size. |
| Hepatitis C virus | **Kamal 2004 [25]** | Prospective cohort | Egypt | Human, Individuals with acute HCV at baseline, with and without schistosome infection | *S. mansoni* | 68 | Coinfected individuals exhibited intrahepatic HCV-specific Th1 responses that were less frequent and smaller magnitude than those with HCV monoinfection. Liver fibrosis worsened more during follow-up in coinfected patients, and was inversely correlated with a Th1 immune response. | This study only examined a limited number of cytokines due to limited sample available. |
| Hepatitis C virus | **El-Kady 2005 [26]** | Cross-sectional | Egypt | Human, Individuals with HCV, individuals with schistosome infection, and individuals with HCV and schistosome infection | *S. mansoni* | 70 | Patients coinfected with HCV and *S. mansoni* had significantly lower serum levels of IFN-gamma and IL-18, and higher levels of IL-4 and IL-10, compared to patients monoinfected with HCV. | Higher HCV RNA reported in co-infection, but numbers not included. This study assumes that HCV was acquired after *S. mansoni*, but the temporal order is unknown. |
| Hepatitis C virus | **Emam 2006 [27]** | Cross-sectional | Egypt | Human, Individuals with chronic HCV with and without schistosome infection | *S. mansoni* | 50 | Individuals coinfected with HCV and *S. mansoni* had significantly lower serum levels of IFN-gamma and higher IL-4 and IL-10 compared to individuals mono-infected with HCV. Those with co-infection also had more severe histologic liver disease. | Not all patients had liver biopsies, which could have introduced bias into the comparison of histologic liver disease severity. |
| Hepatitis C virus | **Ahmed 2016 [28]** | Cross-sectional | Egypt | Human, Individuals with HCV with and without schistosome infection, plus healthy controls | *S. mansoni* | 107 | Schistosome-HCV coinfected individuals had higher HCV RNA viral loads, elevated transaminases, and increased IL-28B but no difference in IFN-g compared to HCV-monoinfected individuals. | The full results are not presented, and there is no description of how the control group was selected. Minimal description of demographic or baseline characteristics. |
| Hepatitis C virus | **Elrefaei 2004 [29]** | Cross-sectional | Egypt | Human, Individuals with chronic HCV infection with and without schistosome infection | *S. mansoni* | 38 | HCV-schistosome coinfected individuals had decreased IFN-gamma and increased IL-10 compared to HCV monoinfected individuals. No difference seen in HCV RNA viral load. No difference in IFN-gamma production in response to HCV peptide stimulation. | This study had a small sample size. Also, this study used only IFN-gamma as a marker of HCV immune activity. |
| Hepatitis C virus | **Bahgat 2010 [30]** | *In vitro* study of human immortal-ized cells | Egypt | Cells with and without *S. mansoni* infection | *S. mansoni* | Not reported | Concentration of HCV antigens was higher and detection of viral RNA was earlier in HepG2 cells infected with *S. mansoni* than those uninfected. | This was an in vitro study; a mouse model would provide further insights but is not possible for HCV-*S. mansoni* coinfection. |
| Hepatitis C virus | **El-Awady 2006 [31]** | *In vitro* study of human PBMCs | Egypt | Individuals with detectable HCV antibodies | *S. haematobium* | 26 | PBMCs cultured with soluble egg antigen of *S. haematobium* had an increased intracellular HCV viral load and increased number of viral strands compared to PBMCs not cultured with *S. haematobium* egg antigens. | Only 3 of 26 people studied were women, and descriptions of statistical analyses are not complete. |
| Hepatotropic virus | **Edwards 2005 [32]** | Mouse model using lymphocytic choriomeningitis virus plus *S. mansoni* | NA | Mice with hepatotropic LCMV virus with and without schistosome infection | *S. mansoni* | Not reported | Mice with *S. manson*i had increased viral replication in the liver compared to uninfected mice. This correlated with a decreased type 1 IFN response. | Mouse LCMV model may have significant differences from human hepatic viral infection. |
| Hepatitis C virus | **El-Shazly 1994 [33]** | Prospective cohort | Egypt | Human, Individuals with HCV with and without schistosome infection | *S. mansoni* | 60 | Following treatment with interferon therapy, coinfected patients had a lower percentage of return to normal alanine aminotrasnferase levels, a lower response rate to interferon therapy after 6 months, and a higher relapse rate than HCV mono-infected patients. | This study did not assess the effects of antischistosomal treatment on interferon therapy. |
| Hepatitis C virus | **Kamal 2000 [34]** | Prospective cohort | Egypt | Human, Individuals with chronic HCV with and without schistosome infection | *S. mansoni* | 62 | Schistosome-HCV coinfected individuals had a lower response rate and higher relapse rate to interferon therapy than HCV-monoinfected individuals. | It is not clear whether investigators controlled for other causes of lower response to interferon therapy in the analysis. |
| Hepatitis C virus | **Attallah 2016 [35]** | Cross-sectional | Egypt | Human, Individuals with HCV with and without schistosome infection | *S. mansoni* | 174 | Schistosome-infected individuals had more severe liver fibrosis than schistosome-uninfected individuals. | The *S. mansoni*-HCV coinfected group contained significantly more women than the group with HCV alone. |
| Hepatitis B virus | **Loffredo-Verde 2020 [36]** | Mouse viral challenge study | NA | Female C57BL/6J and BALB/c mice infected at different time points with schistosomes and acute and chronic HBV | *S. mansoni* | Not reported | HBV viral replication was suppressed during the acute and chronic phases of schistosome infection and liver damage mitigated by T regs during chronic schistosome infection. Suppression of HBV was dependent on IFN-ɣ. | Mouse model requires vector transfer of HBV genomes because HBV infection does not occur in mice. Only female mice used. |
| Hepatitis B virus | **Kotkat 1990 [37]** | Cross-sectional, then prospective cohort | Egypt | Human, Individuals with schistosome infection before and after treatment | *S. mansoni* | 336 | Parasitologically cured individuals were more likely to clear HCV infection than those who remained schistosome infected after treatment. | There was a very small sample size in the prospective portion of this study. |
| Hepatitis B virus | **Farghaly 1993 [38]** | Prospective cohort | Egypt | Human, Individuals with schistosome infection and HBV before and after praziquantel treatment | Not listed | 92 | Coinfected individuals had decreased liver transaminases and more frequently cleared HbSAg post-treatment than those with HBV alone. | This study only included men. |
| Hepatitis B virus | **Chen 2012 [39]** | Mouse vaccine study | NA | Male BALB/c mice with and without schistosome infection, before and after praziquantel treatment | *S. japonicum* | 140 | Mice with *S. japonicum* had signficantly lower anti-HBs titers after HBV vaccination than those without. Anti-HBs titers increased after praziquantel treatment. | Only male mice were used. Humans may regain Th1 immune responses over longer time periods than were required for mice. |
| Hepatitis B virus | **Ghaffar 1990 [40]** | Prospective cohort | Egypt | Human, Boys aged 8-12 with and without schistosome infection | *S. mansoni* | 80 | Schistosome-infected children had lower post-vaccine anti-HBs levels than schistosome uninfected. | Only males were studied. |
| Hepatitis B virus | **Riner 2016 [41]** | Prospective cohort | Kenya | Human, Adults with and without schistosome infection | *S. mansoni* | 146 | In *S. mansoni* infected adults, titers were lower two weeks after the second hepatitis dose and trended toward remaining lower after the third dose. | Only 63% of patients initially enrolled completed follow-up. |
| Hepatitis B virus | **Bassily 1992 [42]** | Prospective cohort | Egypt | Human, 6-12 year olds with and without schistosome infection | *S. mansoni* | 508 | No difference between anti-HB titers in children with and without schistosome infection. Significantly lower titers in children with hepatosplenomegaly. | Very few children had heavy infections; the study may have lacked power to detect an effect that differed by intensity of schistosome infections. |
| Hepatitis B virus | **Bassily 1997 [43]** | Prospective cohort | Egypt | Human, Infants of mothers who have and do not have active schistosome infection | *S. mansoni* | 385 | No difference anti-HBs titers after vaccination, or rates of seroconversion, between infants with mothers with and without *S. mansoni*. | Confounders such as infant health status and other maternal/infant infections were not considered. |
| Hepatitis B virus | **Nash 2017 [44]** | Randomized controlled trial | Uganda | Human, Infants of mothers treated with praziquantel, infants of mothers not treated with praziquantel | *S. mansoni* | 1379 | No effect of maternal praziquantel treatment on infant anti-HBs titers. | Only 55% of eligible infants were included in this study; single stool used to assess *S. mansoni* infection status may have led to misclassification. |
| Hepatitis B virus | **Malhotra 2015 [45]** | Prospective cohort | Kenya | Human, Mothers with and without schistosome infection and their newborns | *S. haematobium* | 450 | Maternal schistosome infection had no effect on anti-HBs antibody titers. | This study used both microscopy and serology to test for schistosome infection, which may have led to misclassification of cases. Vaccine responses were measured after 6 months of age, so immediate response and effect of intervening infections are not known. |
| Hepatitis B virus | **Malhotra 2018 [46]** | Prospective cohort | Kenya | Human, Mothers with and without schistosome infection and their newborns | *S. haematobium* | 450 | Prenatal schistosome exposure did not significantly affect anti-HBs antibody titers or longitudinal trajectories of titers over 30 months. | Impacts of confounders such as malnutrition not addressed and the effects of natural infections on antibody titers was not assessed. |
| HIV | **Kleppa 2014 [47]** | Nested case-control | South Africa | Human, Sexually active females with and without schistosome infection before and after praziquantel treatment | *S. haematobium* | 44 | *S. haematobium* was associated with higher frequencies of CD14+ cells and CD4+ cells expressing CCR5. After treatment, the CD14+ cell population and expression of CCR5 decreased. | The samples in this study were analyzed in two batches eight months apart. Sample size was limited and statistical corrections for multiple comparisons were not used. |
| HIV | **Yegorov 2019 [48]** | Cross-sectional | Uganda | Human, HIV uninfected adult women with and without schistosome infection, before and after treatment. | *S. mansoni* | 36 | HIV viral entry into cervical CD4+ cells was higher in *S. mansoni*-infected women and decreased after praziquantel treatment. Treatment of *S. mansoni* infection also partially normalized dysregulated anti-viral interferon signaling and increased IFN-α. | This study had a small sample size and did not follow patients beyond two months. |
| HIV | **Dupnik 2019 [49]** | Cross-sectional | Tanzania | Human, Women with and without schistosome infection | *S. haematobium & S. mansoni* | 97 | Women with *S. haematobium* had lower levels of IL-15 compared with women without *S. haematobium*. No differences were observed between women with and without *S. mansoni*. | This study had a small sample size, and therefore potential confounders could not be controlled for in analysis. Additionally, this study did not include pre- or post-infection samples, so changes in gene-expression or cytokine levels could not be observed. |
| HIV | **Prodger 2015 [50]** | Cross-sectional | Uganda | Human, HIV-uninfected men with and without schistosome infection | *S. mansoni* | 34 | *S. mansoni* infection was associated with increased frequencies of Th1, Th17, and Th22 in blood but not foreskin. | This study had a small sample size. They also did not test for other sexually transmitted infections beyond HSV-2 that could have altered foreskin immune cell populations. |
| HIV | **Secor 2003 [51]** | Cross-sectional | Kenya | Human, HIV-infected men with active schistosome infection and HIV-infected men previously treated for schistosome infection | *S. mansoni* | 42 | Coinfected men had higher densities of HIV co-receptors CXCR4 and CCR5 on the surface of CD4+ T cells than men who had been previously treated, and co-receptor density decreased after praziquantel treatment. | Only men were studied. Densities of HIV co-receptors were not compared to those who had never had schistosome infection but only to those previously treated but still actively exposed to schistosome infection. |
| HIV | **McElroy 2005 [52]** | Cross-sectional | Uganda | Human, HIV-infected adults with and without schistosome infection | *S. mansoni* | 35 | HIV-schistosome coinfected individuals had decreased Gag-specific CD8+ cytolytic T-cell responses and increased Gag-specific IL-10-positive CD8+ T-cell responses compared to HIV mono-infected. | This study had a small sample size. The cross-sectional design does not allow controlling for all confounders, though matching on several important cofactors was helpful. |
| HIV | **Erikstrup 2008 [53]** | Cross-sectional with open randomization for prospective follow-up | Zimbabwe | Human, HIV-infected individuals with and without schistosome infection, before and after treatment | *S. haematobium & S. mansoni* | 378 | IL-8 was increased in *S. mansoni* infected participants. After being treated with praziquantel, STNF-rII and IL-8 both decreased. | Study could only be carried out for 3 months for ethical reasons (some patients were randomized to delayed praziquantel). The confounding effects of other infections not assessed. |
| HIV | **Obuku 2016 [54]** | Cross-sectional | Uganda | Human, HIV-infected individuals with and without schistosome infection | *S. mansoni* | 50 | There were no differences between viral load and CD4 between HIV-schistosome coinfected individuals and HIV monoinfected individuals. Coinfected individuals had increased TNF-α after non-viral stimulation and increased CD8+IFN-ɣ+ and CD4+IFN-ɣ+TNF-α+ T cells after Gag stimulation. | This study had a small sample size. Individuals had recently acquired HIV and they may not be comparable to people with more chronic HIV infection. |
| HIV | **Dzhivhuho 2018 [55]** | Mouse vaccine study | NA | Mice with schistosome infection before and after praziquantel treatment; mice without schistosome infection | *S. mansoni* | Not reported | Mice with schistosome infection had lower-magnitude HIV-specific CD8+ T-cell responses to HIV vaccines than mice without schistosome infection. Mice given praziquantel treatment before vaccination had lower titers of gp140-specific IgG and only partial restoration of CD4+ and CD8+ T-cell responses 12 days after vaccination. | Immunological effects of HIV vaccination on schistosome infected and uninfected mice are incompletely elucidated in mouse models. |
| HIV | **Actor 1993 [56]** | Mouse viral challenge study | NA | 10-12 week-old female BALB/c mice with and without schistosome infection given recombinant vaccina virus vPE16 | *S. mansoni* | Not reported | Mice with schistosome infection produced minimal IFN-ɣ and IL-2 in response to viral challenge with recombinant vaccinia virus expressing the HIV glycoprotein gp160. Schistosome-infected mice also experienced decreased CD8+ cytotoxicity and delayed viral clearance. | All mice were female. |
| HIV | **Marshall 2001 [57]** | Mouse coinfection model | NA | Female BALB/c mice with and without schistosome infection given vaccinia virus | *S. mansoni* | Not reported | A non-T, non-B, non-NK cell actively suppresses cytotoxic activity during vaccinia virus infection in mice with *S. mansoni.* | Only female mice were used. |
| HIV | **Actor 1994 [58]** | Mouse viral challenge study | NA | 10-12 week-old female BALB/c mice with recombinant vaccina virus vPE16 with and without schistosome infection | *S. mansoni* | Not reported | Delayed viral clearance in schistosome-infected mice only occurred after the onset of worm egg-laying and was associated with egg granuloma formation. | All mice were female. Also, difficult to determine whether behavior of vaccinia virus in mouse hepatic granulomas is reflective of human hepatic viruses. |
| HIV | **Lacroix 1998 [59]** | Mouse coinfection model | NA | Female LP-BM5-infected C57BL/6 mice with and without schistosome infection | *S. mansoni* | Not reported | Murine AIDS did not progress faster in mice with *S. mansoni.*Schistosome-infected mice had increased IL-4 compared to uninfected mice 21 weeks after infection. | Only female mice were used in this study. No statistical correction for multiple comparisons was performed. |
| HIV | **Chenine 2005 [60]** | Macaque coinfection model | NA | Macaques with and without schistosome infection that were given simian HIV infection | *S. mansoni* | 8 | Rhesus macaques coinfected with sHIV and *S. mansoni* had increased expression of Th2 -associated cytokines and increased sHIV viral replication compared to sHIV-monoinfected macaques. | Only a single high dose of SHIV was tested. Also, macaques spontaneously clear *S. mansoni infection* so long-term follow-up is not possible. |
| HIV | **Ayash-Rashkovsky 2007 [61]** | Macaque coinfection model | NA | Simian HIV-infected rhesus macaques, of which some were given schistosome infection | *S. mansoni* | 15 | Macaques with sHIV that were given *S. mansoni* had significantly higher mRNA expression of Th2 cytokine response, reactivation of previously suppressed sHIV, and sustained decreased frequency of CD4+CD29+ memory T cells compared to those with sHIV alone. | Macaques naturally clear *S. mansoni* infection and this could impact viral dynamics. |
| HIV | **Buch 2001 [62]** | Macaque coinfection model | NA | Macaques with chronic sHIV with *S. mansoni* eggs injected into liver and lungs or complete Freund's adjuvant challenge injected intradermally | *S. mansoni* | 8 | Macaques with sHIV that were given *S. mansoni* had tissue macrophages that permitted sHIV viral replication and increased IL-4. Macaques that received CFA had tissue macrophages without viral replication and had tissue IL-4, IL-10, IFN-ɣ, and IL-2 levels detected. | Small study, and tissue macrophages in skin may differ from those in liver and lung. |
| HIV | **Mouser 2019 [64]** | *In vitro* study of human peripheral blood mononuclear cells | NA | Cells that matured in the presence or absence of schistosome soluble egg antigen in various immune environments | *S. mansoni* | Not reported | There was increased resistance to trans-infection in HIV-susceptible human dendritic cells that matured while exposed to schistosome egg antigens in a Th2-predominant environment compared to cells that matured in a Th1 environment. | IL-2 could not be studied because it is a necessary part of the cell culture system. |
| HIV | **Kjetland 2006 [65]** | Cross-sectional | Zimbabwe | Human, Sexually active women with and without schistosome infection | *S. haematobium* | 527 | Women with S. haematobium had a three-fold increased risk for HIV. | Because this was a cross-sectional study, causality could not be assessed. |
| HIV | **Downs 2011 [66]** | Cross-sectional | Tanzania | Human, Reproductive-aged women with and without schistosome infection | *S. haematobium & S. mansoni* | 457 | Women with schistosome infection had higher rates of HIV than women without schistosome infection. | Single urine and stool samples were used, decreasing the sensitivity for detection of schistosome infections. Also, as a cross-sectional study it is unable to determine causality. |
| HIV | **Downs 2012 [67]** | Cross-sectional | Tanzania | Human, Reproductive-aged women with and without schistosome infection | Predominantly *S. mansoni* | 345 | Schistosome infection was strongly associated with HIV. | As a cross-sectional study, this was unable to determine causality. Use of schistosome antigen testing precluded identification of the species of schistosome infection. |
| HIV | **Mvumbi 2018 [68]** | Cross-sectional | Demo-cratic Republic of Congo | Human, Women with and without HIV and *S. haematobium* infection | *S. haematobium* | 446 | The prevalence of HIV was 28% among women with *S. haematobium* infection (23/81) versus 11% among women without (40/365). | Abstract only; no adjustments for clinical or demographic criteria reported. |
| HIV | **Downs 2017 (Am J Trop Med Hyg) [69]** | Cross-sectional | Tanzania | Human, Adult men with and without schistosome infection | *S. haematobium & S. mansoni* | 674 | Men with schistosome infection did not have a higher odds of HIV infection than men without schistosome infection. | This study did not have the power to detect an increased odds of HIV infection that was less than 2.0. |
| HIV | **Woodburn 2009 [70]** | Cross-sectional | Uganda | Human, Pregnant women screened for helminths and HIV infection | *S. mansoni* | 2507 | No significant increase in odds of HIV among women with *S. mansoni* infection | Relied on egg excretion, which is known to be lower in women and those with HIV |
| HIV | **Sanya 2015 [71]** | Cross-sectional | Uganda | Human, Individuals with and without schistosome infection | *S. mansoni* | 1,412 | There was no association between HIV and *S. mansoni*. | This was a cross-sectional study in a fishing community with intense exposure to *S. mansoni* and risky sexual behavior. Other known risks for HIV were not assessed or controlled for. |
| HIV | **Mazigo 2014 [72]** | Cross-sectional | Tanzania | Human, Adults with and without schistosome infection | *S. mansoni* | 1,785 | There was no difference in HIV prevalence between *S. mansoni*-infected and -uninfected individuals. | This study was cross-sectional and used single stool samples, yielding decreased sensitivity. Analysis of HIV prevalence was not separated by sex. |
| HIV | **De Lima e Costa 1988 [73]** | Cross-sectional | Brazil | Human, stored serum from people with *S. mansoni* infection status known were tested for HIV | *S. mansoni* | 180 | The prevalence of HIV did not differ between those with and without *S. mansoni* infection, although only 5 sera were reactive. | Likely underpowered given very low prevalence of HIV. |
| HIV | **Ssetaala 2015 [74]** | Nested case-control | Uganda | Human, HIV-seroconverters and HIV-uninfected controls | *S. mansoni* | 200 | *S. mansoni* was not associated with HIV acquisition. However, there was an eight-fold increased odds of HIV infection in those who had not received anti-schistosome treatment in the past two years. | This study did not analyze separately by sex. Receiving anti-schistosome praziquantel treatment in the past two years was self-reported. |
| HIV | **Bochner 2020 [75]** | Nested case-control | Kenya and Uganda | Human, HIV-seroconverters and HIV-uninfected controls at high risk for HIV acquisition had blood samples tested for schistosome antigen | *S. haematobium & S. mansoni* | 2250 | No significant increase in odds of HIV acquisition was noted in either sex for schistosome infections overall or for either schistosome species individually. | Power to detect increased risk for *S. haematobium* was limited. Both were high-risk cohorts and adjustment for STIs, which differed between seroconverters and controls, was not performed. |
| HIV | **Kroidl 2016 [76]** | Prospective cohort | Tanzania | Human, Community-based cohort with known *S. haematobium* infection status were followed for incident HIV infection | *S. haematobium* | 1055 | The risk ratio for incident HIV was not increased in people who had *S. haematobium* eggs visualized at some point in the study. | The study was observational so may have been influenced by confounders. The timecourse of treatment of the *S. haematobium* infections is unclear. |
| HIV | **Downs 2017 (PLoS Negl Trop Dis) [77]** | Nested case-control | Tanzania | Human, Adults with and without schistosome infection | *S. haematobium & S. mansoni* | 338 | Women with schistosome infection had an increased odds of HIV acquisition compared to women without schistosome infection, but there was no difference in men. Median plasma viral load was higher in those with HIV-schistosome coinfection than those with HIV alone. | This study does not distinguish between schistosome species. This study was also unable to test for other helminth infections and unable to assure that no participants were taking ART, though at the time of the study ART was very scarcely available in the region and only for CD4<200 and pregnant mothers. |
| HIV | **Gallagher 2005 [78]** | Retros-pective cohort | Kenya | Human, Mothers with and without schistosome infection and their infants | *S. haematobium* | 936 | Mother-to-child HIV transmission was not different between schistosome infected and uninfected mothers. However, the presence of any helminth infection (including schistosome infection) was associated with increased mother-to-child HIV transmission. | This was a retrospective cohort and important other factors associated with mother-to-child HIV transmission were not included in the study, including maternal HIV viral load, concurrent sexually transmitted infections, and other factors at delivery. |
| HIV | **Chenine 2008 [79]** | Macaque coinfection model | NA | Macaques with and without schistosome infection that were given simian HIV infection via rectal inoculation | *S. mansoni* | 17 | Schistosome-infected macaques were infected with SHIV at viral doses that were 17-fold lower than the doses necessary to infect schistosome-uninfected macaques. HIV schistosome coinfected macaques also had higher peak viral RNA loads and increased CD4+ viral replication. | The macaque model system only allows studies during acute schistosome infection because macaques self-clear infection by 20-25 weeks. |
| HIV | **Siddappa 2011 [80]** | Macaque coinfection model | NA | Macaques with and without schistosome infection that were given simian HIV intravenously | *S. mansoni* | 16 | There was no difference in SHIV dose needed to achieve infection when schistosome-infected and -uninfected macaques were infected intravenously. | This study had a small sample size and therefore may have lacked power to detect smaller but significant differences in SHIV doses required to establish infection via intravenous inoculation. |
| HIV | **Marti 2017 [86]** | Cross-sectional | Tanzania | Human, HIV-infected adults on ART with and without schistosome infection | *S. mansoni* | 305 | Schistosome-HIV coinfection was associated with increased liver transaminases. | This study did not differentiate between schistosome species but assumed all cases were *S. mansoni.* The study controlled for many, but not all, causes of elevated transaminases and was cross-sectional so not able to determine causality. |
| HIV | **Midzi 2017 [87]** | Prospective cohort | Zimbabwe | Human, HIV-infected men with schistosome infection who were and were not on ART | *S. haematobium* | 18 | Among 6 men not on ART, HIV-1 RNA viral load in both plasma and semen trended towards a decrease from baseline to a timepoint 10 weeks after praziquantel treatment. | This study had a small sample size and findings did not reach significance. Also, the study did not control for other factors that could affect viral load such as other infections, and no men without schistosome infections were included. |
| HIV | **Brown 2004 [90]** | Prospective cohort | Uganda | Human, HIV-infected individuals with and without schistosome infection | *S. mansoni* | 429 | There was no difference in CD4 count between schistosome-coinfected and HIV-monoinfected individuals. Those coinfected had higher viral loads than those monoinfected. Praziquantel treatment did not affect viral load. | Schistosome-infected participants were all treated and then analyzed as those with versus without persistent schistosome infection, which may obscure effects of other variables such as recent schistosome reinfection. |
| HIV | **Kleppa 2015 [91]** | Cross-sectional | South Africa | Human, HIV-infected and -uninfected girls with and without schistosome infection | *S. haematobium* | 792 | There was no association between CD4 count and *S. haematobium* in HIV-infected or HIV-uninfectedwomen. | Women were not tested for other helminth infections or STIs, which could also influence CD4 counts. Only a single urine sample was tested. |
| HIV | **Colombe 2018 (Front Immunol) [92]** | Cross-sectional | Tanzania | Human, HIV-infected adults with and without schistosome infection | *S. haematobium* & *S. mansoni* | 83 | There was no diference in viral load between schistosome-infected and -uninfected adults. | This study had a small sample size and did not account for other possible co-infections that could alter viral load. |
| HIV | **Colombe 2018 (PLoS Negl Trop Dis) [93]** | Prospective cohort | Tanzania | Human, HIV-infected adults with and without schistosome infection | *S. haematobium* & *S. mansoni* | 172 | Schistosome-infected adults were less likely to develop CD4 counts <350 cells/ul or death than schistosome-uninfected adults. | Sero-surveys were only collected every 3 years. This study did not test viral load. Follow-up was limited to those who sought care at HIV clinics in the catchment area. |
| HIV | **Idindili 2011 [94]** | Cross-sectional | Tanzania | Human, HIV-infected adults with and without schistosome infection | Not stated | 464 | There was no difference in CD4+ T cell counts between those with and without schistosome infection. | Cross-sectional design and could not determine causality. The study also did not control for other causes of CD4+ T cell count changes including duration of HIV infection and concurrent other infections. |
| HIV | **Elliott 2003 [95]** | Prospective cohort | Uganda | Human, HIV-infected adults with and without schistosome infection, before and after praziquantel treatment | *S. mansoni* | 108 | Coinfected individuals had higher CD4 counts than HIV-monoinfected individuals. There was no difference in CD4 count before and 12 weeks after praziquantel treatment. Four to five weeks after treatment, viral load increased, but these increases did not persist 4-5 months after treatment. | This study had a small sample size. The baseline analysis was cross-sectional and higher CD4 counts could not be causally attributed to schistosome infection. The study also did not examine differences between those treated with praziquantel and those not treated. |
| HIV | **Efraim 2013 [96]** | Retros-pective cohort | Tanzania | Human, HIV-infected adults on ART with and without schistosome infection | *S. haematobium & S. mansoni* | 351 | Individuals with HIV-schistosome coinfection had lower increases in CD4 counts following ART initiation than HIV-monoinfected individuals. | This study did not test HIV viral loads and could not determine whether impairments in CD4 increases were associated with elevated viral loads. Also, the retrospective study increases the possibility of bias in the study population included. |
| HIV | **Mulu 2013 [97]** | Prospective cohort | Ethiopia | Human, HIV-infected adults with and without schistosome infection, before and after praziquantel treatment | *S. mansoni* | 220 | There was no difference in CD4 count before and 12 weeks after praziquantel treatment. | This study had a small sample size. Only a very small fraction of the patients originally screened completed follow-up. |
| HIV | **Mazigo 2016 [98]** | Prospective cohort | Tanzania | Human, HIV-infected adults with and without schistosome infection, before and after praziquantel treatment | *S. mansoni* | 50 | There was no association between schistosome infection and CD4 count, and CD4 count did not change after treatment. Viral loads were significantly higher among coinfected individuals compared to HIV mono-infected individuals. Treatment did not affect viral load. | This was a small study and participants were only followed for 3 months after praziquantel treatment. Only 12 of 22 participants treated for *S. mansoni* completed follow-up. |
| HIV | **Abaasa 2018 [99]** | Randomized controlled trial | Uganda | Human, ART naïve HIV-infected individuals with and without schistosome infection, before and after praziquantel treatment | *S. mansoni* | 363 | There was no difference in CD4 count in those given praziquantel treatment quarterly compared to annually, but those treated quarterly trended towards higher viral loads. The group treated quarterly also trended toward increased risk for progression to AIDS. | Some ART use occurred during the trial; also there was a high predominance of males given the conduct of this trial in fishing villages. |
| HIV | **Brown 2005 [100]** | Prospective cohort | Uganda | Human, HIV-schistosome coinfected individuals before and after praziquantel treatment | *S. mansoni* | 163 | Individuals successfully treated for *S. mansoni* infection had lower CD4+ counts six months after treatment than those still infected. Those successfully treated also had increases in viral load 4-5 weeks after treatment that were not sustained 4-5 months after treatment. | The schistosome infection status was not assessed at follow-up and the possibility of re-infection causing the effects seen at 4-5 months cannot be ruled out. |
| HIV | **Bochner 2019 [101]** | Prospective cohorts (4) | Kenya, Uganda | Human, Recent HIV-seroconverters with and without schistosome infection | *S. haematobium* & *S. mansoni* | 370 | *S. mansoni* infection was associated with a decline in plasma HIV viral loads and *S. haematobium* was associated with a decline in cervical HIV viral loads. | Schistosome status was measured only once prior to HIV acquisition. |
| HIV | **Masikini 2019 [102]** | Case-control | Tanzania | Human, HIV-infected adults failing ART with and without schistosome infection | *S. haematobium & S. mansoni* | 188 | There was no association between schistosome infection and viral load. | The cohort in this study had overall high viral loads because of immunological failure. The inclusion of patients with immunological failure only may make this study less generalizable. |
| HIV | **Kallestrup 2005 [103]** | Randomized controlled trial | Zimbabwe | Human, HIV-schistosome coinfected adults who received immediate or delayed praziquantel treatment | *S. haematobium & S. mansoni* | 227 | Individuals who received praziquantel treatment delayed three months after baseline had higher viral loads than those who received praziquantel treatment at baseline. | This study could only be carried out for 3 months for ethical reasons. It was not blinded. |
| HIV | **Lawn 2000 [104]** | Prospective cohort | Kenya | Human, Individuals with HIV-schistosome coinfection before and after treatment | *S. mansoni* | 30 | Viral load increased after effective treatment of schistosome-HIV coinfection, particularly in those who were followed up six or more months post-treatment. | This study also did not have HIV positive age and sex-matched controls without schistosome infection due to the high community prevalence of infection, and did not have untreated people for comparison due to ethical reasons. The possibility of other infections impacting viral load was incompletely addressed. |
| HIV | **Stete 2018 [105]** | Retros-pective cohort | Tanzania | Human, HIV-infected individuals with and without schistosome infection | *S. haematobium & S. mansoni* | 461 | There was a hazard ratio of 0.58 [0.32-1.05] for death or loss to follow-up in those with HIV-schistosome coinfection. | Clinical data was collected prospectively but CAA was examined retrospectively and schistosome species was not known. Confounding factors could influence results. |
| HIV | **Da'dara 2006 [106]** | Mouse vaccine study | NA | Female 6-8 week old BALB/c mice with and without schistosome infection | *S. mansoni* | Not reported | Schistosome-infected mice had decreased HIV-specific CD8+ T-cell IFN-ɣ secretion after HIV vaccination compared to uninfected mice. | Only female mice were used in this study. |
| HIV | **Da'dara 2010 [107]** | Mouse vaccine study | NA | Female 6-8 week old BALB/c mice with schistosome infection, with schistosome infection that was treated, and without schistosome infection | *S. mansoni* | Not reported | HIV-specific CD8+ T-cell IFN-ɣ secretion was increased in mice that had schistosome infections treated prior to receiving the HIV vaccine. T-cell responses were partially restored when vaccine was administered four weeks after treatment, and completely restored when vaccine was given 8 weeks after treatment. | Only female mice were used in this study. |
| Gamma herpesvirus | **Reese 2014 [108]** | Mouse coinfection model | NA | C57BL6/J mice with chronic murine herpesvirus that were and were not given *S. mansoni* coinfection | *S. mansoni* | Not reported | Mice with chronic latent murine herpesvirus that were given *S. mansoni* infection developed systemic herpesvirus reactivation with concomitant increases in IL-4 and decreased IFN-gamma compared to herpesvirus mono-infected mice. | Some experiments were performed for both *S. mansoni* and *H. polygyrus,* while others were performed for *H. polygyrus* only. |
| Kaposi's sarcoma-associated herpesvirus | **Fu 2012 [110]** | Cross-sectional | China | Human, Individuals with and without schistosome infection | *S. japonicum* | 105 | *S. japonicum*-infected men had a higher KSHV seroprevalence than uninfected men (8.4 vs 2.8%). This difference was not observed in women. Overall KSHV seroprevalence was 8.4% in *S. japonicum* infected versus 6.6% in uninfected. | Did not assess or control for HIV coinfection. Also, because this was a cross-sectional study, causality could not be assessed. |
| Kaposi's sarcoma-associated herpesvirus | **Wakeham 2011 [111]** | Cross-sectional | Uganda | Human, pregnant women tested for helminths and KSHV antibodies | *S. mansoni* | 1,915 | No difference in KSHV seroprevalence between women with and without *Sm* in a peri-urban setting. | Cross-sectional design was unable to assess causality. It is also possible that pregnancy affected antibody levels. |
| Kaposi's sarcoma-associated herpesvirus | **Nalwoga 2019 [112]** | Cross-sectional with longitudinal follow-up | Uganda | Human, Individuals with and without schistosome infection in fishing communities | *S. mansoni* | 1,137 | *S. mansoni*-infected individuals were more frequently KSHV-seropositive (89% versus 77%) with increased odds of seropositivity after controlling for age, sex, HIV, and other parasitic infections. | Because this was a cross-sectional study, causality could not be assessed. |
| Murid gammaherpesvirus 4 (MuHV-4) | **Rolot 2018 [113]** | Mouse coinfection model | NA | Female BALB/c mice with acute respiratory herpesvirus with and without schistosome infection | *S. mansoni* | Not reported | Coinfected mice had increased IL-4 and expanded virus-specific CD8+ T-cell effector responses in the lung, leading to increased control of the acute respiratory virus. | None listed. |
| Influenza A | **Danz 2016 [114]** | Mouse coinfection model | NA | Mice with and without schistosome infection | *S. mansoni* | Not reported | Mice with schistosome infection had decreased mortality, decreased proportions of influenza-specific interferon gamma positive CD8+ T cells, decreased pro-inflammatory response to influenza A, and decreased alveolar inflammation compared to mice without schistosome infection. | Only female mice were used. |
| Influenza A | **Tundup 2017 [115]** | Mouse coinfection model | NA | Mice with and without schistosome infection | *S. mansoni* | Not reported | Mice with chronic schistosome infection that were exposed to influenza A had decreased lung injury, weight loss, and pro-inflammatory cytokine gene expression compared to mice with influenza A alone. | Only the abstract is available, making full assessment of results and the quality of the study challenging. |
| Influenza A | **Kadl 2018 [116]** | Mouse coinfection model | NA | Mice with and without schistosome infection | *S. mansoni* | Not reported | Mice with chronic schistosome infection that were exposed to influenza A had increased epithelial regeneration, improved survival, and less weight loss that appeared to be mediated by Type I Interferon receptor and IL-4. Mice with *S. mansoni* also had increased influenza-specific antibodies and CD8+ T cell responses. | Only the abstract is available, making full assessment of results and the quality of the study challenging. |
| Influenza A, Pneumonia virus of mice | **Scheer 2014 [117]** | Mouse coinfection model | NA | Mice with and without schistosome infection | *S. mansoni* | Not reported | Mice with chronic schistosome infection were significantly protected from a viral respiratory challenge compared to mice without schistosome infection. Protection correlated with the onset of TNF-alpha mediated goblet cell hyperplasia and mucus secretion. | The model of mouse *S. mansoni* infection used is less commonly used and does not induce hepatosplenomegaly syndrome, raising questions about the generalizability of their findings. |
| Human papillomavirus | **Kjetland 2010 [119]** | Follow-up after a cross-sectional study | Zimb-abwe | Human, Women with high-risk HPV five years earlier, with and without schistosome infection | *S. haematobium* | 37 | Increased frequency of high-grade squamous intraepithelial lesion (HGSIL) in women with *S. haematobium.* No difference in HPV persistence. | This study had a small sample size and women were tested for high-risk HPV only twice over a long time span. 15% of women had died and could not be assessed. |
| Human papillomavirus | **Petry 2003 [120]** | Cross-sectional | Tanz-ania/ Germ-any | Human, Women with and without schistosome infection | *S. haematobium* | 218 | There was a borderline difference in prevalence of detectable genital HPV DNA between schistosome-infected and -uninfected women, but only 6 women had confirmed *S. haematobium.* | The study grouped women with reported history of anti-schistosome treatment together with those with confirmed active infection. When women with confirmed infection were analyzed alone, no significant differences were seen. Also, women from Germany used as a control population may have other differences that affect genital HPV DNA aside from schistosome infection status. |
| Human papillomavirus | **Gent 2019 [121]** | Baboon vaccine study | NA | Olive baboons with and without schistosome infection before and after HPV vaccination | *S. mansoni* | 10 | *S. mansoni*-infected baboons had lower HPV-specific IgG titers than uninfected baboons. | This study had a very small sample size. |
| Human papillomavirus | **Brown 2014 [122]** | Prospective cohort | Tanzania | Human, Adolescent girls and young women with and without schistosome infection | *S. haematobium* & *S. mansoni* | 298 | There was no difference in anti-HPV antibody titers between schistosome-infected and uninfected individuals 7 and 12 months after vaccination. | This study is observational, so results may be influenced by confounders. |
| HTLV-1 | **Porto 2005 [124]** | Cross-sectional | Brazil | Human, HTLV-1-infected individuals with and without schistosome infection | *S. mansoni* | 342 for epidemiologic study; 70 for immunologic study | Coinfection was associated with decreased IFN-ɣ and increased IL-10 and IL-5. Intestinal helminth infections were seven-fold more frequent in HTLV-1 disease carriers than in HTLV-1 infected persons who developed tropical spastic paralysis. | Participants had *Strongyloides stercoralis* and/or *S. mansoni* infection so effects of *S. mansoni* alone cannot be ascertained. |
| HTLV-1 | **Lima 2013 [125]** | Cross-sectional | Brazil | PBMCs from HTLV-1-infected individuals (carriers and HAM-TSP) for cytokine stimulation studies | *S. mansoni* | 26 | Stimulation with schistosome antigens led to decreased IFN-gamma and increased IL-10 in measurements of cytokines from stimulated versus unstimulated PBMC cultures. | The study did not control for other infections or patient characteristics that could affect the responses to stimulation in PBMCs. Also, no patients without HTLV-1 were included as controls. |
| HTLV-1 | **Lima 2017 [126]** | Cross-sectional | Brazil | PBMCs from HTLV-1-infected individuals and healthy controls for cytokine stimulation studies | *S. mansoni* | 38 | The addition of *S. mansoni* antigens to PBMC cultures from HTLV-1-infected adults caused downregulation of CXCL9. | The healthy controls were much younger than others but due to the small sample size this and other differences may not have reached statistical significance. |
| HTLV-1 | **Santos 2004 [127]** | Cross-sectional | Brazil | Human, HTLV-1-infected individuals with and without schistosome or strongyloides infection | *S. mansoni* | 70 for the relevant portion of the study | Helminth-HTLV-1 coinfected individuals had decreased IFN-ɣ and increased IL-10 in unstimulated PBMC cultures compared to those with HTLV-1 alone. | The study lacks descriptions of the demographic characteristics of participants and how the controls were selected beyond being matched for age and sex. Data is not reported for *S. mansoni* separate from *S. stercoralis* infection. |
| Measles | **Tweyon-gyere 2019 [128]** | Randomized trial | Uganda | Human, Children with and without schistosome infection | *S. mansoni* | 239 | *S. mansoni-*infected children who received a catch-up measles vaccination achieved lower anti-measles IgG titers. Praziquantel treatment restored these titers, but children still infected at follow-up continued to have decreased titers. | Participants were followed up at limited time points. Long-term effects of untreated schistosome infections could not be studied for ethical reasons. |
| Measles | **Nono 2018 [129]** | Cross-sectional | Cam-eroon | Human, Children with and without schistosome infection | *S. haematobium & S. mansoni* | 525 total; 66 had anti-measles titers | Anti-measles antibodies were significantly lower in children infected with *S. mansoni* compared to egg-negative controls. | This study included children with symptoms other that schistosome infection that could be affecting outcomes. Also, measles vaccination status was ascertained by parental report. |
| Measles | **Jiz 2013 [130]** | Cross-sectional | Philip-pines | Human, Children with and without schistosome infection | Not listed | 104 | Children with schistosome infections had similarly robust cytokine responses to measles antigen stimulation post-vaccination compared to children without schistosome infections. | Only the abstract is available, making full assessment of results and the quality of the study challenging. Also, most children had at least one helminth infection. |
| Measles | **Ondigo 2018 [131]** | Prospective cohort | Kenya | Human, Mothers with and without schistosome infection and their infants | *S. mansoni* | 99 | Two-year-old children born to mothers with *S. mansoni* had lower anti-measles IgG titers compared to children born to uninfected mothers. | Schistosome infection status of the children was not assessed, nor were other demographic or clinical characteristics of the children. |
| Measles | **Kizito 2013 [132]** | Observ-ational cohort within a randomized controlled trial | Uganda | Human, Mothers with and without schistosome infection and their infants | *S. mansoni* | 711 | Maternal helminth infection duirng pregnancy does not influence levels of measles-specific antibodies in infants up to one year old. | In this study, they did not collect information on maternal measles immune status. Also, schistosome infection status of the infants was not known. |
| Measles | **Storey 2017 [133]** | Cross-sectional | Kenya | Human, HIV-infected ART-naïve adults with and without schistosome and other helminth infections | *S. mansoni* | 100 | Anti-measles IgG titers were similar regardless of schistosome infection status. | Only a small number of participants (16) had schistosome infection and power to detect a difference was limited. |
