# Supplementary material for: Effects of schistosomes on host anti-viral immune response and the acquisition, virulence, and prevention of viral infections: A systematic review
Source: PLoS Pathog. 2021 May 20;17(5):e1009555. doi: 10.1371/journal.ppat.1009555 (PMC8172021; doi:10.1371/journal.ppat.1009555)
Supplement: S1 Text — (DOCX) [file ppat.1009555.s003.docx]

**S1 Text. Full Search Strategy.**

## **OVID MEDLINE® ALL Complete Search Strategy**

The following is the comprehensive search strategy for the systematic review. It was first performed on March 28, 2019 and was re-run on December 9, 2019 using OVID MEDLINE® ALL.

| 1. Viruses/ |  |
| --- | --- |
| 2. Virology/ |  |
| 3. HIV Infections/ |  |
| 4. HIV-1/ |  |
| 5. Macaca mulatta/vi [Virology] |  |
| 6. Mucous Membrane/vi [Virology] |  |
| 7. Simian Acquired Immunodeficiency Syndrome/ |  |
| 8. Simian Immunodeficiency Virus/ |  |
| 9. Viral Load/ |  |
| 10. Virus Replication/ |  |
| 11. Herpesviridae/ |  |
| 12. Herpesviridae Infections/ |  |
| 13. Herpesvirus 8, Human/ |  |
| 14. Virus Activation/ |  |
| 15. Virus Latency/ |  |
| 16. Vaccinia virus/ |  |
| 17. HIV Envelope Protein gp160/ |  |
| 18. Vaccines/ |  |
| 19. Vaccination/ |  |
| 20. Acquired Immunodeficiency Syndrome/ |  |
| 21. HIV Seroprevalence/ |  |
| 22. HIV Seropositivity/ |  |
| 23. RNA, Viral/ |  |
| 24. Enterovirus/ |  |
| 25. Enterovirus Infections/ |  |
| 26. Feces/vi [Virology] |  |
| 27. Coinfection/ |  |
| 28. Hepacivirus/ |  |
| 29. Virus Diseases/ |  |
| 30. Hepatitis C, Chronic/ |  |
| 31. Liver/vi [Virology] |  |
| 32. Hepatitis C/ |  |
| 33. Hepatomegaly/vi [Virology] |  |
| 34. Splenomegaly/vi [Virology] |  |
| 35. Viremia/ |  |
| 36. Caliciviridae Infections/ |  |
| 37. Gastroenteritis/vi [Virology] |  |
| 38. Intestines/vi [Virology] |  |
| 39. Norovirus/ |  |
| 40. HTLV-I Infections/ |  |
| 41. Human T-lymphotropic virus 1/ |  |
| 42. Deltaretrovirus Infections/ |  |
| 43. (Virus* or Zoophaginae).mp. [mp=title, abstract, original title, name of substance word, subject heading word, floating sub-heading word, keyword heading word, organism supplementary concept word, protocol supplementary concept word, rare disease supplementary concept word, unique identifier, synonyms] |  |
| 44. Virology.mp. |  |
| 45. (HIV Infection* or HTLV-III-LAV Infection* or HTLV III LAV Infection* or T-Lymphotropic Virus Type III or HTLV-III Infection* or HTLV III Infection* or HIV Coinfection* or HIV Coinfections or hiv seropositivity or human immunodeficiency virus encephalopathy).mp. [mp=title, abstract, original title, name of substance word, subject heading word, floating sub-heading word, keyword heading word, organism supplementary concept word, protocol supplementary concept word, rare disease supplementary concept word, unique identifier, synonyms] |  |
| 46. (Simian AIDS or Simian Acquired Immuno-Deficiency Syndrome or Simian Acquired Immuno Deficiency Syndrome or Simian Acquired Immune Deficiency Syndrome or Simian AIDSs or SAIDS or Simian Acquired Immunodeficiency Syndrome).mp. [mp=title, abstract, original title, name of substance word, subject heading word, floating sub-heading word, keyword heading word, organism supplementary concept word, protocol supplementary concept word, rare disease supplementary concept word, unique identifier, synonyms] |  |
| 47. (Simian Immunodeficiency Virus* or HTLV-4 or HTLV-IV or human T cell lymphotropic virus type 4 or SIV).mp. [mp=title, abstract, original title, name of substance word, subject heading word, floating sub-heading word, keyword heading word, organism supplementary concept word, protocol supplementary concept word, rare disease supplementary concept word, unique identifier, synonyms] |  |
| 48. (Viral Burden or Virus Titer or Viral Load or viral titre or viral titer or virus burden or virus particle count or virus titre or virus load).mp. [mp=title, abstract, original title, name of substance word, subject heading word, floating sub-heading word, keyword heading word, organism supplementary concept word, protocol supplementary concept word, rare disease supplementary concept word, unique identifier, synonyms] |  |
| 49. (Virus Replication* or viral replication or virus reproduction).mp. [mp=title, abstract, original title, name of substance word, subject heading word, floating sub-heading word, keyword heading word, organism supplementary concept word, protocol supplementary concept word, rare disease supplementary concept word, unique identifier, synonyms] |  |
| 50. (Herpesviruses or Thymic Group Virus* or Mouse Thymic Virus* or Murid herpesvirus 3 or herpes virus or herpesvirus or herpesviruses or herpetovirus or Herpesviridae).mp. [mp=title, abstract, original title, name of substance word, subject heading word, floating sub-heading word, keyword heading word, organism supplementary concept word, protocol supplementary concept word, rare disease supplementary concept word, unique identifier, synonyms] |  |
| 51. (Herpesviridae Infection* or Herpesvirus Infection* or B Virus Infection* or Herpesviridae infection* or herpesviral infection* or herpes virus infection).mp. [mp=title, abstract, original title, name of substance word, subject heading word, floating sub-heading word, keyword heading word, organism supplementary concept word, protocol supplementary concept word, rare disease supplementary concept word, unique identifier, synonyms] |  |
| 52. (HHV-8 or KSHV or Kaposi's Sarcoma-Associated Herpesvirus or Kaposi's Sarcoma Associated Herpesvirus or Kaposis Sarcoma-Associated Herpesvirus or Kaposi Sarcoma-Associated Herpesvirus or Kaposi Sarcoma Associated Herpesvirus or Human Herpesvirus 8 or Kaposi's Sarcoma-Associated Herpesviruses or herpes simplex virus 8 or herpes virus 8 or herpes virus type 8 or herpesvirus 8 or human herpes virus 8 or kaposi sarcoma associated herpesvirus).mp. [mp=title, abstract, original title, name of substance word, subject heading word, floating sub-heading word, keyword heading word, organism supplementary concept word, protocol supplementary concept word, rare disease supplementary concept word, unique identifier, synonyms] |  |
| 53. (Virus Activation* or Viral Activation* or Virus Induction* or Prophage Induction* or Prophage Excision*).mp. [mp=title, abstract, original title, name of substance word, subject heading word, floating sub-heading word, keyword heading word, organism supplementary concept word, protocol supplementary concept word, rare disease supplementary concept word, unique identifier, synonyms] |  |
| 54. (Virus Latenc* or Viral Latenc*).mp. [mp=title, abstract, original title, name of substance word, subject heading word, floating sub-heading word, keyword heading word, organism supplementary concept word, protocol supplementary concept word, rare disease supplementary concept word, unique identifier, synonyms] |  |
| 55. (Vaccinia virus* or Poxvirus officinale or Rabbitpox virus* or Rabbit Pox Virus* or Buffalopox virus* or Buffalo Pox Virus* or inactivated vaccinia virus or pox virus officinalis or Vaccinia orthopoxvirus or Vaccinia pox virus or vaccinia variolae virus).mp. [mp=title, abstract, original title, name of substance word, subject heading word, floating sub-heading word, keyword heading word, organism supplementary concept word, protocol supplementary concept word, rare disease supplementary concept word, unique identifier, synonyms] |  |
| 56. (HIV Envelope Protein gp160 or HTLV-III gp160 or HTLV III gp160 or gp160 or HIV Envelope Glycoprotein gp160 or glycoprotein gp160 or gp 160 or hiv envelope protein gp160).mp. [mp=title, abstract, original title, name of substance word, subject heading word, floating sub-heading word, keyword heading word, organism supplementary concept word, protocol supplementary concept word, rare disease supplementary concept word, unique identifier, synonyms] |  |
| 57. (combined vaccine or vaccin* or vaccine control or vaccine efficacy or vaccine potency or vaccine safety).mp. [mp=title, abstract, original title, name of substance word, subject heading word, floating sub-heading word, keyword heading word, organism supplementary concept word, protocol supplementary concept word, rare disease supplementary concept word, unique identifier, synonyms] |  |
| 58. (Vaccination* or Active Immunization* or vaccination program or vaccination programme or vaccinotherapy or virus vaccination).mp. [mp=title, abstract, original title, name of substance word, subject heading word, floating sub-heading word, keyword heading word, organism supplementary concept word, protocol supplementary concept word, rare disease supplementary concept word, unique identifier, synonyms] |  |
| 59. (Acquired Immune Deficiency Syndrome* or Acquired Immuno Deficiency Syndrome* or Acquired Immuno-Deficiency Syndrome* or Acquired Immunodeficiency Syndrome* or AIDS).mp. [mp=title, abstract, original title, name of substance word, subject heading word, floating sub-heading word, keyword heading word, organism supplementary concept word, protocol supplementary concept word, rare disease supplementary concept word, unique identifier, synonyms] |  |
| 60. (HIV Seroprevalence or Human immunodeficiency virus prevalence or hiv prevalence).mp. [mp=title, abstract, original title, name of substance word, subject heading word, floating sub-heading word, keyword heading word, organism supplementary concept word, protocol supplementary concept word, rare disease supplementary concept word, unique identifier, synonyms] |  |
| 61. (HIV Seropositivit* or AIDS Seropositivit* or AIDS Seropositivit* or Anti HIV Positivit* or Anti-HIV Positivit* or HTLV III Seropositivit* or HTLV-III Seropositivit* or HIV Seroconversion* or HTLV-III Seroconversion* or HTLV III Seroconversion* or AIDS Seroconversion* or HIV Antibody Positivit*).mp. [mp=title, abstract, original title, name of substance word, subject heading word, floating sub-heading word, keyword heading word, organism supplementary concept word, protocol supplementary concept word, rare disease supplementary concept word, unique identifier, synonyms] |  |
| 62. (Viral RNA or viral ribonucleic acid or viral rna or virus ribonucleic acid or virus RNA).mp. [mp=title, abstract, original title, name of substance word, subject heading word, floating sub-heading word, keyword heading word, organism supplementary concept word, protocol supplementary concept word, rare disease supplementary concept word, unique identifier, synonyms] |  |
| 63. (Enterovirus or Coxsackie or entero virus or Coxsackieviruses).mp. [mp=title, abstract, original title, name of substance word, subject heading word, floating sub-heading word, keyword heading word, organism supplementary concept word, protocol supplementary concept word, rare disease supplementary concept word, unique identifier, synonyms] |  |
| 64. (Enterovirus Infection* or enteroviral infection*).mp. [mp=title, abstract, original title, name of substance word, subject heading word, floating sub-heading word, keyword heading word, organism supplementary concept word, protocol supplementary concept word, rare disease supplementary concept word, unique identifier, synonyms] |  |
| 65. (Coinfection* or Polymicrobial Infection* or Co-infection* or Mixed Infection* or Secondary Infection* or multiple infection*).mp. [mp=title, abstract, original title, name of substance word, subject heading word, floating sub-heading word, keyword heading word, organism supplementary concept word, protocol supplementary concept word, rare disease supplementary concept word, unique identifier, synonyms] |  |
| 66. (Hepaciviruses or Hepatitis C Like Virus* or Hepatitis C-Like Virus* or Hepatitis C virus*).mp. [mp=title, abstract, original title, name of substance word, subject heading word, floating sub-heading word, keyword heading word, organism supplementary concept word, protocol supplementary concept word, rare disease supplementary concept word, unique identifier, synonyms] |  |
| 67. (arbovirus infection* or tumor virus infection* or tumour virus infection* or viral disease* or viral infection* or virus disease* or Virus Infections or Virus Disease or Virus Infection).mp. [mp=title, abstract, original title, name of substance word, subject heading word, floating sub-heading word, keyword heading word, organism supplementary concept word, protocol supplementary concept word, rare disease supplementary concept word, unique identifier, synonyms] |  |
| 68. Chronic Hepatitis C.mp. |  |
| 69. (Hepatitis C or Parenterally-Transmitted Non-A, Non-B Hepatitis or Parenterally Transmitted Non A, Non B Hepatitis or PT-NANBH or parenterally transmitted non a non b hepatitis).mp. [mp=title, abstract, original title, name of substance word, subject heading word, floating sub-heading word, keyword heading word, organism supplementary concept word, protocol supplementary concept word, rare disease supplementary concept word, unique identifier, synonyms] |  |
| 70. (Viremia* or viraemia).mp. [mp=title, abstract, original title, name of substance word, subject heading word, floating sub-heading word, keyword heading word, organism supplementary concept word, protocol supplementary concept word, rare disease supplementary concept word, unique identifier, synonyms] |  |
| 71. (Caliciviridae Infection* or Calicivirus Infection* or Norovirus Infection*).mp. [mp=title, abstract, original title, name of substance word, subject heading word, floating sub-heading word, keyword heading word, organism supplementary concept word, protocol supplementary concept word, rare disease supplementary concept word, unique identifier, synonyms] |  |
| 72. (Norovirus* or Norwalk-like Virus* or Norwalk like Virus* or Small Round-Structured Virus* or Small Round Structured Virus*).mp. [mp=title, abstract, original title, name of substance word, subject heading word, floating sub-heading word, keyword heading word, organism supplementary concept word, protocol supplementary concept word, rare disease supplementary concept word, unique identifier, synonyms] |  |
| 73. (HTLV I Infection* or Human T-lymphotropic Virus 1 Infection* or Human T lymphotropic Virus 1 Infection* or HTLV-I Infection* or Human T cell leukemia virus I infection*).mp. [mp=title, abstract, original title, name of substance word, subject heading word, floating sub-heading word, keyword heading word, organism supplementary concept word, protocol supplementary concept word, rare disease supplementary concept word, unique identifier, synonyms] |  |
| 74. (Human T lymphotropic virus 1 or Adult T-Cell Leukemia-Lymphoma Virus I or ATLV or HTLV-I or Human T Cell Leukemia Virus I or HTLV-1 or Human T-Cell Leukemia Virus I or Adult T Cell Leukemia Lymphoma Virus I).mp. [mp=title, abstract, original title, name of substance word, subject heading word, floating sub-heading word, keyword heading word, organism supplementary concept word, protocol supplementary concept word, rare disease supplementary concept word, unique identifier, synonyms] |  |
| 75. (Deltaretrovirus Infection* or HTLV-BLV Infection* or HTLV BLV Infection* or HTLV Infection* or BLV Infection* or deltaretroviral infection).mp. [mp=title, abstract, original title, name of substance word, subject heading word, floating sub-heading word, keyword heading word, organism supplementary concept word, protocol supplementary concept word, rare disease supplementary concept word, unique identifier, synonyms] |  |
| 76. 1 or 2 or 3 or 4 or 5 or 6 or 7 or 8 or 9 or 10 or 11 or 12 or 13 or 14 or 15 or 16 or 17 or 18 or 19 or 20 or 21 or 22 or 23 or 24 or 25 or 26 or 27 or 28 or 29 or 30 or 31 or 32 or 33 or 34 or 35 or 36 or 37 or 38 or 39 or 40 or 41 or 42 or 43 or 44 or 45 or 46 or 47 or 48 or 49 or 50 or 51 or 52 or 53 or 54 or 55 or 56 or 57 or 58 or 59 or 60 or 61 or 62 or 63 or 64 or 65 or 66 or 67 or 68 or 69 or 70 or 71 or 72 or 73 or 74 or 75 |  |
| 77. Schistosoma/ |  |
| 78. Schistosomiasis/ |  |
| 79. Schistosoma mansoni/ |  |
| 80. Schistosomiasis mansoni/ |  |
| 81. Schistosoma haematobium/ |  |
| 82. Schistosomiasis haematobia/ |  |
| 83. Schistosoma japonicum/ |  |
| 84. Schistosomiasis japonica/ |  |
| 85. (Schistosoma* or Bilharzia*).mp. [mp=title, abstract, original title, name of substance word, subject heading word, floating sub-heading word, keyword heading word, organism supplementary concept word, protocol supplementary concept word, rare disease supplementary concept word, unique identifier, synonyms] |  |
| 86. Schistosoma incognitum.mp. |  |
| 87. (Schistosomias* or Schistoma Infection* or Katayama Fever or Bilharzias* or bilharziasis or bilharziosis or schistomiasis or Schistosoma infection or schistosomatosis or schistosome infection or schistosomosis).mp. [mp=title, abstract, original title, name of substance word, subject heading word, floating sub-heading word, keyword heading word, organism supplementary concept word, protocol supplementary concept word, rare disease supplementary concept word, unique identifier, synonyms] |  |
| 88. Schistosoma manson*.mp. |  |
| 89. (Schistosoma mansoni Infection* or Intestinal Schistosomiases or Intestinal Schistosomiasis or bilharziasis mansoni or schistosomiasis mansoni).mp. [mp=title, abstract, original title, name of substance word, subject heading word, floating sub-heading word, keyword heading word, organism supplementary concept word, protocol supplementary concept word, rare disease supplementary concept word, unique identifier, synonyms] |  |
| 90. (Schistosoma haematobium* or Bilharzia hematobium or Schistoma haematobium or Bilharzia haematobia).mp. [mp=title, abstract, original title, name of substance word, subject heading word, floating sub-heading word, keyword heading word, organism supplementary concept word, protocol supplementary concept word, rare disease supplementary concept word, unique identifier, synonyms] |  |
| 91. (Schistosoma haematobia Infection* or Schistosomiasis haematobi* or Urinary Schistosomias* or Urogenital Schistosomias* or Schistosoma haematobium infection* or Schistosoma hematobium infection* or schistosomiasis haematobium or schistosomiasis hematobia or schistosomiasis hematobium).mp. [mp=title, abstract, original title, name of substance word, subject heading word, floating sub-heading word, keyword heading word, organism supplementary concept word, protocol supplementary concept word, rare disease supplementary concept word, unique identifier, synonyms] |  |
| 92. (Schistosoma japonicum* or Schistosoma japonica or Schistosoma cattoi or Schistosoma japonicus).mp. [mp=title, abstract, original title, name of substance word, subject heading word, floating sub-heading word, keyword heading word, organism supplementary concept word, protocol supplementary concept word, rare disease supplementary concept word, unique identifier, synonyms] |  |
| 93. (Schistosomiasis japonic* or Schistosoma japonicum Infection* or Schistosoma japonica infection or schistosomiasis japonicum).mp. [mp=title, abstract, original title, name of substance word, subject heading word, floating sub-heading word, keyword heading word, organism supplementary concept word, protocol supplementary concept word, rare disease supplementary concept word, unique identifier, synonyms] |  |
| 94. 77 or 78 or 79 or 80 or 81 or 82 or 83 or 84 or 85 or 86 or 87 or 88 or 89 or 90 or 91 or 92 or 93 |  |
| 95. CD4-Positive T-Lymphocytes/ |  |
| 96. Disease Susceptibility/ |  |
| 97. Mucous Membrane/im [Immunology] |  |
| 98. Schistosomiasis mansoni/im [Immunology] |  |
| 99. Schistosoma mansoni/im [Immunology] |  |
| 100. Simian Acquired Immunodeficiency Syndrome/im [Immunology] |  |
| 101. Interferon-gamma/ |  |
| 102. Interleukin-4/ |  |
| 103. CD8 Antigens/ |  |
| 104. Cytotoxicity, Immunologic/im [Immunology] |  |
| 105. HIV-1/im [Immunology] |  |
| 106. Immunity, Cellular/ |  |
| 107. Lung/im [Immunology] |  |
| 108. Spleen/im [Immunology] |  |
| 109. T-Lymphocyte Subsets/ |  |
| 110. T-Lymphocytes, Cytotoxic/ |  |
| 111. Vaccinia virus/im [Immunology] |  |
| 112. Vaccines/im [Immunology] |  |
| 113. Biomarkers/ |  |
| 114. HIV Infections/co [Complications] |  |
| 115. Schistosomiasis/co [Complications] |  |
| 116. Antigens, Helminth/ |  |
| 117. HIV Infections/im [Immunology] |  |
| 118. Schistosoma haematobium/im [Immunology] |  |
| 119. Immunity, Mucosal/ |  |
| 120. Schistosomiasis/im [Immunology] |  |
| 121. Viral Load/im [Immunology] |  |
| 122. HIV/im [Immunology] |  |
| 123. Intestinal Diseases/im [Immunology] |  |
| 124. Malnutrition/im [Immunology] |  |
| 125. Helminthiasis/co [Complications] |  |
| 126. Intestinal Diseases, Parasitic/co [Complications] |  |
| 127. Leukocytes, Mononuclear/ |  |
| 128. Lymphocyte Activation/ |  |
| 129. Tetanus Toxoid/im [Immunology] |  |
| 130. Th1 Cells/ |  |
| 131. Th2 Cells/ |  |
| 132. Coinfection/im [Immunology] |  |
| 133. Hepatitis C, Chronic/im [Immunology] |  |
| 134. Interleukin-8/ |  |
| 135. T-Lymphocytes, Regulatory/ |  |
| 136. Morbidity/ |  |
| 137. CD4 Antigens/ |  |
| 138. Eosinophilia/ |  |
| 139. Integrin beta1/ |  |
| 140. Lymphocyte Subsets/ |  |
| 141. CD8-Positive T-Lymphocytes/ |  |
| 142. Caliciviridae Infections/im [Immunology] |  |
| 143. Coinfection/im [Immunology] |  |
| 144. Gastroenteritis/im [Immunology] |  |
| 145. Immunomodulation/ |  |
| 146. Intestines/im [Immunology] |  |
| 147. Macrophage Activation/ |  |
| 148. Macrophages/ |  |
| 149. Norovirus/im [Immunology] |  |
| 150. Carrier State/im [Immunology] |  |
| 151. HTLV-I Infections/co, im [Complications, Immunology] |  |
| 152. Interleukin-10/ |  |
| 153. Interleukin-5/ |  |
| 154. Paraparesis, Tropical Spastic/co, im [Complications, Immunology] |  |
| 155. Deltaretrovirus Infections/im [Immunology] |  |
| 156. Down-Regulation/im [Immunology] |  |
| 157. Human T-lymphotropic virus 1/im [Immunology] |  |
| 158. Neuroschistosomiasis/ |  |
| 159. Monocytes/ |  |
| 160. Genitalia, Female/im [Immunology] |  |
| 161. Genital Diseases, Female/im [Immunology] |  |
| 162. (CD4 Positive T Lymphocyte* or CD4-Positive T-Lymphocyte* or T4 Cell* or T4 Lymphocyte* or CD4 T cell* or CD4 T lymphocyte* or CD4+ T cell* or CD4+ T lymphocyte* or CD4-positive T-cell*).mp. [mp=title, abstract, original title, name of substance word, subject heading word, floating sub-heading word, keyword heading word, organism supplementary concept word, protocol supplementary concept word, rare disease supplementary concept word, unique identifier, synonyms] |  |
| 163. (Diathes* or Disease Susceptibilit* or disease proneness or familial predisposition or predisposing factor or predisposition).mp. [mp=title, abstract, original title, name of substance word, subject heading word, floating sub-heading word, keyword heading word, organism supplementary concept word, protocol supplementary concept word, rare disease supplementary concept word, unique identifier, synonyms] |  |
| 164. (gamma-Interferon or Immune Interferon or Type II Interferon or Interferon-gammahuman immune interferon or ifn gamma or human immune interferon or imunomax gamma or interferon 2 or interferon gamma or interferon ii or interferon type II or interferon-gamma or oh 6000).mp. [mp=title, abstract, original title, name of substance word, subject heading word, floating sub-heading word, keyword heading word, organism supplementary concept word, protocol supplementary concept word, rare disease supplementary concept word, unique identifier, synonyms] |  |
| 165. (Interleukin-4 or Interleukin 4 or B-Cell Growth Factor-1 or B Cell Growth Factor 1 or B-Cell Growth Factor-I or B Cell Growth Factor I or B-Cell Proliferating Factor or B Cell Proliferating Factor or B-Cell Stimulating Factor-1 or B Cell Stimulating Factor 1 or B-Cell Stimulatory Factor 1 or B-Cell Stimulatory Factor-1 or BCGF-1 or Binetrakin or BSF-1 or IL-4 or IL4 or Mast Cell Growth Factor-2 or Mast Cell Growth Factor 2 or MCGF-2 or B Cell Stimulatory Factor-1 or B Cell Stimulatory Factor b cell stimulating factor or B cell stimulatory factor 1 or b cell stimulatory factor i or b lymphocyte stimulating factor 1 or bsf 1 or bsf1 or eosinophil differentiation factor or il 4).mp. [mp=title, abstract, original title, name of substance word, subject heading word, floating sub-heading word, keyword heading word, organism supplementary concept word, protocol supplementary concept word, rare disease supplementary concept word, unique identifier, synonyms] |  |
| 166. (T-Cell T8 Antigens or T Cell T8 Antigens or Leu-2 Antigens or Leu 2 Antigens or CD8 Antigen* or antigen cd8 or antigen cd 8 or cd 8 antigen or cd8).mp. [mp=title, abstract, original title, name of substance word, subject heading word, floating sub-heading word, keyword heading word, organism supplementary concept word, protocol supplementary concept word, rare disease supplementary concept word, unique identifier, synonyms] |  |
| 167. (Cellular Immunit* or Cell Mediated Immunit* or Cell-Mediated Immunit* or Cellular Immune Response*adoptive immunity or cell immunity or cell mediated immune response or cell mediated immunity or cellular defence or cellular immune reaction or cellular immune response or adoptive immunity or T cell immunity or t lymphocyte immunity).mp. [mp=title, abstract, original title, name of substance word, subject heading word, floating sub-heading word, keyword heading word, organism supplementary concept word, protocol supplementary concept word, rare disease supplementary concept word, unique identifier, synonyms] |  |
| 168. (T Lymphocyte Subset* or T-Lymphocyte Subset* or T-Cell Subset* or T Cell Subset* or T cell subpopulation* or t cell subset or T lymphocyte subpopulations or T lymphocytic subpopulation or T lymphocytic subset or T cell subpopulation).mp. [mp=title, abstract, original title, name of substance word, subject heading word, floating sub-heading word, keyword heading word, organism supplementary concept word, protocol supplementary concept word, rare disease supplementary concept word, unique identifier, synonyms] |  |
| 169. (Cell Mediated Lympholytic Cell* or Cell-Mediated Lympholytic Cell* or Cytotoxic T-Lymphocyte* or Cytotoxic T Lymphocyte* or TC2 Cell* or TC1 Cell* or t cell cytotoxicity or T lymphocyte cytotoxicity).mp. [mp=title, abstract, original title, name of substance word, subject heading word, floating sub-heading word, keyword heading word, organism supplementary concept word, protocol supplementary concept word, rare disease supplementary concept word, unique identifier, synonyms] |  |
| 170. (Biologic Marker* or Biological Marker* or Laboratory Marker* or Serum Marker* or Surrogate Endpoint* or Surrogate End Point* or Clinical Marker* or Viral Marker* or Biochemical Marker* or Immune Marker* or Immunologic Marker* or Surrogate Marker*).mp. [mp=title, abstract, original title, name of substance word, subject heading word, floating sub-heading word, keyword heading word, organism supplementary concept word, protocol supplementary concept word, rare disease supplementary concept word, unique identifier, synonyms] |  |
| 171. Helminth Antigen*.mp. |  |
| 172. (Mucosal Immunity or Mucosal Immune Respons*).mp. [mp=title, abstract, original title, name of substance word, subject heading word, floating sub-heading word, keyword heading word, organism supplementary concept word, protocol supplementary concept word, rare disease supplementary concept word, unique identifier, synonyms] |  |
| 173. (Mononuclear Leukocyte* or mononuclear cell*).mp. [mp=title, abstract, original title, name of substance word, subject heading word, floating sub-heading word, keyword heading word, organism supplementary concept word, protocol supplementary concept word, rare disease supplementary concept word, unique identifier, synonyms] |  |
| 174. (immune cell clone or Blast Transformation or Blastogenesis or Lymphocyte Stimulation or Lymphocyte Transformation or Lymphoblast Transformation).mp. [mp=title, abstract, original title, name of substance word, subject heading word, floating sub-heading word, keyword heading word, organism supplementary concept word, protocol supplementary concept word, rare disease supplementary concept word, unique identifier, synonyms] |  |
| 175. (Th1 Cell* or TH-1 Cell* or TH 1 Cell* or T Helper 1 Cell* or Type 1 Helper T Cell* or T H 1 cell or T helper 1 or T helper type 1 or T-cell helper type 1 or T-cell helper-1 or T-helper cell type 1 or helper cell type 1).mp. [mp=title, abstract, original title, name of substance word, subject heading word, floating sub-heading word, keyword heading word, organism supplementary concept word, protocol supplementary concept word, rare disease supplementary concept word, unique identifier, synonyms] |  |
| 176. (Th2 Cell* or T Helper 2 Cell* or TH 2 Cell* or TH-2 Cell* or T Helper2 Cell* or Type-2 Helper T Cell* or Type 2 Helper T Cell* or T helper type 2 or T helper 2 or helper cell type 2).mp. [mp=title, abstract, original title, name of substance word, subject heading word, floating sub-heading word, keyword heading word, organism supplementary concept word, protocol supplementary concept word, rare disease supplementary concept word, unique identifier, synonyms] |  |
| 177. (Interleukin 8 or IL8 or Monocyte-Derived Neutrophil Chemotactic Factor or Neutrophil Activation Factor or Lymphocyte-Derived Neutrophil-Activating Peptide or Monocyte-Derived Neutrophil-Activating Peptide or Alveolar Macrophage Chemotactic Factor-I or Alveolar Macrophage Chemotactic Factor I or AMCF-I or Anionic Neutrophil-Activating Peptide or Anionic Neutrophil Activating Peptide or Chemokine CXCL8 or CXCL8 Chemokines or Macrophage-Derived Chemotactic Factor or Neutrophil Chemotactic Factor or CXCL8 Chemokine or Granulocyte Chemotactic Peptide-Interleukin-8 or Granulocyte Chemotactic Peptide Interleukin 8 or IL-8chemokine CXCL8 or CXC chemokine ligand 8 or CXCL8 chemokine or granulocyte chemotactic peptide or il 8 or lymphocyte derived neutrophil activating peptide or lynap or monap or monocyte derived neutrophil activating peptide or monocyte derived neutrophil chemotactic factor or neutrophil activating factor or neutrophil activating peptide or neutrophil attracting peptide or polymorphonuclear granulocyte activating factor).mp. [mp=title, abstract, original title, name of substance word, subject heading word, floating sub-heading word, keyword heading word, organism supplementary concept word, protocol supplementary concept word, rare disease supplementary concept word, unique identifier, synonyms] |  |
| 178. (Regulatory T Lymphocyte* or Regulatory T-Lymphocyte* or Regulatory T-Cell* or Regulatory T Cell* or Treg Cell* or Th3 Cell* or Naturally-Occurring Suppressor T-Lymphocyte* or Naturally-Occurring Suppressor T-Cell* or Tr1 Cell* or immunoregulatory T cell or immunoregulatory T cells or immunoregulatory T lymphocyte or T regulatory cell or T regulatory cells or T regulatory lymphocyte or Treg or Tregs).mp. [mp=title, abstract, original title, name of substance word, subject heading word, floating sub-heading word, keyword heading word, organism supplementary concept word, protocol supplementary concept word, rare disease supplementary concept word, unique identifier, synonyms] |  |
| 179. (Morbidit* or disease incidence or disorder incidence or disease frequency).mp. [mp=title, abstract, original title, name of substance word, subject heading word, floating sub-heading word, keyword heading word, organism supplementary concept word, protocol supplementary concept word, rare disease supplementary concept word, unique identifier, synonyms] |  |
| 180. (CD4 Molecule* or T-Cell T4 Antigen* or Surface CD4 Receptor* or CD4 Antigen* or CD4 Receptor* or antigen cd4 or antigen cd 4 or cd 4 antigen or cd4 or cd4 glycoprotein or glycoprotein cd 4 or glycoprotein cd4).mp. [mp=title, abstract, original title, name of substance word, subject heading word, floating sub-heading word, keyword heading word, organism supplementary concept word, protocol supplementary concept word, rare disease supplementary concept word, unique identifier, synonyms] |  |
| 181. (Eosinophilia* or Tropical Eosinophilia* or eosinophilosis or eosinophylia or eosinophilic leukocytosis).mp. [mp=title, abstract, original title, name of substance word, subject heading word, floating sub-heading word, keyword heading word, organism supplementary concept word, protocol supplementary concept word, rare disease supplementary concept word, unique identifier, synonyms] |  |
| 182. (CD29 Antigen* or beta1 Integrin or 4B4 Antigen or CDw29 Antigen or antigen cd29 or CD29 antigen or glycoprotein 2a or glycoprotein IIa or integrin beta 1 or integrin beta1 or beta 1 integrin).mp. [mp=title, abstract, original title, name of substance word, subject heading word, floating sub-heading word, keyword heading word, organism supplementary concept word, protocol supplementary concept word, rare disease supplementary concept word, unique identifier, synonyms] |  |
| 183. (Lymphocyte Subset or Lymphocyte Subpopulation* or lymphocytic subpopulation or lymphocytic subset).mp. [mp=title, abstract, original title, name of substance word, subject heading word, floating sub-heading word, keyword heading word, organism supplementary concept word, protocol supplementary concept word, rare disease supplementary concept word, unique identifier, synonyms] |  |
| 184. (CD8 Positive T Lymphocyte* or T8 Lymphocyte* or T8 Cell* or CD8-Positive Lymphocyte* or CD8-Positive Lymphocyte* or CD8-Positive Suppressor T-Lymphocyte* or CD8-Positive Suppressor T-Cell* or CD8 T cell* or CD8 T lymphocyte* or CD8+ T cell* or CD8+ T lymphocytes or CD8-positive T-cell* or CD8-positive T-lymphocyte*).mp. [mp=title, abstract, original title, name of substance word, subject heading word, floating sub-heading word, keyword heading word, organism supplementary concept word, protocol supplementary concept word, rare disease supplementary concept word, unique identifier, synonyms] |  |
| 185. (Immunomodulation* or Immunomodulatory Therapy or neuroimmunomodulation).mp. [mp=title, abstract, original title, name of substance word, subject heading word, floating sub-heading word, keyword heading word, organism supplementary concept word, protocol supplementary concept word, rare disease supplementary concept word, unique identifier, synonyms] |  |
| 186. Macrophage Activation*.mp. |  |
| 187. (Bone Marrow-Derived Macrophage* or Bone Marrow Derived Macrophage* or Monocyte-Derived Macrophage* or Monocyte Derived Macrophage* or Macrophage* or Monocyte-Derived Macrophage* or clasmatocyte or macrophagocytic cell).mp. [mp=title, abstract, original title, name of substance word, subject heading word, floating sub-heading word, keyword heading word, organism supplementary concept word, protocol supplementary concept word, rare disease supplementary concept word, unique identifier, synonyms] |  |
| 188. (Interleukin-10 or IL10 or IL-10 or CSIF-10 or Cytokine Synthesis Inhibitory Factor or Interleukin 10 or csif).mp. [mp=title, abstract, original title, name of substance word, subject heading word, floating sub-heading word, keyword heading word, organism supplementary concept word, protocol supplementary concept word, rare disease supplementary concept word, unique identifier, synonyms] |  |
| 189. (Interleukin 5 or BCGF-II or T-Cell-Replacing Factor or T Cell Replacing Factor or IL-5 or IL5 or T-Cell Replacing Factor or B-Cell Growth Factor-II or B Cell Growth Factor II or Eosinophil Differentiation Factor or b cell growth factor ii or il 5 or interleukin-5 killer helper factor or T cell replacing factor or T lymphocyte replacing factor or b cell growth factor 2 or killer helper factor).mp. [mp=title, abstract, original title, name of substance word, subject heading word, floating sub-heading word, keyword heading word, organism supplementary concept word, protocol supplementary concept word, rare disease supplementary concept word, unique identifier, synonyms] |  |
| 190. (Neuroschistosomias* or Central Nervous System Schistosomiasis or Schistosomal Myeloradiculopathy or Schistosomal Myeloradiculopathies or Schistosomal Myelitis or Schistosomal Myelopathy or Schistosomal Myelopathies or cerebral biharziasis or cerebral schistosomiasis or CNS schistosomiasis).mp. [mp=title, abstract, original title, name of substance word, subject heading word, floating sub-heading word, keyword heading word, organism supplementary concept word, protocol supplementary concept word, rare disease supplementary concept word, unique identifier, synonyms] |  |
| 191. Monocyte*.mp. |  |
| 192. 95 or 96 or 97 or 98 or 99 or 100 or 101 or 102 or 103 or 104 or 105 or 106 or 107 or 108 or 109 or 110 or 111 or 112 or 113 or 114 or 115 or 116 or 117 or 118 or 119 or 120 or 121 or 122 or 123 or 124 or 125 or 126 or 127 or 128 or 129 or 130 or 131 or 132 or 133 or 134 or 135 or 136 or 137 or 138 or 139 or 140 or 141 or 142 or 143 or 144 or 145 or 146 or 147 or 148 or 149 or 150 or 151 or 152 or 153 or 154 or 155 or 156 or 157 or 158 or 159 or 160 or 161 or 162 or 163 or 164 or 165 or 166 or 167 or 168 or 169 or 170 or 171 or 172 or 173 or 174 or 175 or 176 or 177 or 178 or 179 or 180 or 181 or 182 or 183 or 184 or 185 or 186 or 187 or 188 or 189 or 190 or 191 |  |
| 193. 76 and 94 and 192 |  |
| 194. Immunity, Innate/ |  |
| 195. (immune system* or host reactivity or host response or immun* or Natural Resistance).mp. [mp=title, abstract, original title, name of substance word, subject heading word, floating sub-heading word, keyword heading word, organism supplementary concept word, protocol supplementary concept word, rare disease supplementary concept word, unique identifier, synonyms] |  |
| 196. 192 or 194 or 195 |  |
| 197. 76 and 94 and 196 |  |

## **Grey Literature Search Strategy**

We did a grey literature search with no date restrictions during August and September 2020. Grey literature was assessed through a search of government websites for countries where schistosomiasis is endemic, schistosomiasis focused websites and organizations, neglected tropical disease websites and organizations, professional associations on infectious and/or tropical diseases, and clinical trial registries. We additional performed a Google Scholar search for theses focused on schistosomiasis and viral co-infection. When available, the most recent published manuscripts derived from any applicable grey literature sources were included within our final analysis.
