# Supplementary material for: Effects of schistosomes on host anti-viral immune response and the acquisition, virulence, and prevention of viral infections: A systematic review
Source: PLoS Pathog. 2021 May 20;17(5):e1009555. doi: 10.1371/journal.ppat.1009555 (PMC8172021; doi:10.1371/journal.ppat.1009555)
Supplement: S2 Text — (DOCX) [file ppat.1009555.s004.docx]

**S2. Decision rules for data selection and extraction processes**

**Eligibility criteria for inclusion**

Studies of humans and animals with schistosome infection (any species) and some interaction with a virus, with comparator groups that are likely to be humans or animals without schistosome infection, or those that have been treated for schistosome infection.

**Exclusion criteria**

Studies were excluded if they did not meet the above eligibility criteria or fell into at least one of the following 11 categories:

1. Review pieces, opinion pieces, case studies, and case series (including studies in which extremely low numbers of schistosome infections were identified and therefore statistical analysis was not performed)
2. Outcomes not related to control or prevention of virus
3. CAA testing or egg excretion not used for diagnosis of schistosomiasis, due to wanting to confirm active infection
4. Examined coinfection with non-viruses
5. Included in Abruzzi review, which was very comprehensive through 2015
6. Duplicate
7. Population was entirely schistosomiasis infected
8. Not available in English
9. Presents a study protocol, not results
10. Unable to be obtained
11. Abstract without enough information and no full paper
