# Supplementary material for: Effects of schistosomes on host anti-viral immune response and the acquisition, virulence, and prevention of viral infections: A systematic review
Source: PLoS Pathog. 2021 May 20;17(5):e1009555. doi: 10.1371/journal.ppat.1009555 (PMC8172021; doi:10.1371/journal.ppat.1009555)
Supplement: Attachment — Submitted filename: Review_ResponseToReviewers_Final.docx [file ppat.1009555.s005.docx]

Dear Reviewers,

Thank you for your thoughtful comments on our manuscript, *Effects of Schistosomes on Host Anti-Viral Immune Response and the Acquisition, Virulence, and Prevention of Viral Infections: a Systematic Review*. We have carefully incorporated your feedback into our revised manuscript. Below you will find individual responses to your comments.

**Reviewer 1**

*The only issue approaching major status is the need to proceed with greater caution on suggestions that schistosomiasis coinfection may be beneficial with respect to some viral infections as this may lead to excuses not to execute the WHO 2030 roadmap that expands praziquantel access and distribution. In a few situations (e.g., HTLV), the immunomodulatory effects of chronic schistosomiasis may decrease the pathology caused by the viral infection. However, it is not ethically or logistically tenable that treatment should be withheld from certain individuals. A more nuanced way to approach this possibility would be to encourage research into the factors that are beneficial (e.g., what aspect of schistosome infections increase Treg cells) and employ these separately from any possibility that treatment would be denied to some individuals. Specifically, decrease in HIV viral load should be removed from the "beneficial" side of Figure 2. The data that the authors present in the paper from references 97 and 103 is far from robust--it is not even statistically significant. A much stronger body of literature would be needed to justify inclusion on that side of the balance. It would be fine however to note that the data are equivocal--perhaps moving the viral load designation over the fulcrum is more appropriate. Again, I do not think the authors would want a control program using this figure to justify failure to distribute praziquantel.*

Thank you for this comment. We agree that it is not ethically or logistically tenable for treatment to be withheld and that further nuanced language would benefit the manuscript. We have added emphasis for the need for further research on this topic in the abstract and fourth paragraph of the discussion. Additionally, we have modified Figure 2, indicating that the decrease of viral load is only “possible” and including a footnote indicating that some studies did not reach statistical significance and further research is needed.

*There are a number of minor issues to be addressed. Mainly they are related to being clear about which comparisons are being made. For example, towards the end of page 7, the authors state that people with HBV-S. mansoni coinfections who receive praziquantel treatment more frequently cleared serum HbSAg than those with HBV (infection) alone. It seems like the more appropriate comparison would be in the coninfected individuals before and after treatment rather than between treated individuals and those who never had schistosomiasis to begin with. Please clarify.*

Thank you for bringing our attention to these points. We agree with the reviewer and have addressed each of these individual points below in order to add clarity to the manuscript. On this point, we appreciate the helpful comment and have revised the text to report the pre-post comparison seen in the HBV-*S. mansoni* coinfected individuals before and after praziquantel treatment.  *Similarly, the last sentence of the paragraph at the top of page 9 suggests that treatment with praziquantel could improve vaccine responsiveness and decrease global HBV burden but this is only true in people that had previously had schistosomiasis. Certainly this is implied and the authors probably wanted to avoid being pedantic but as a standalone sentence it makes no sense and the clause "in persons with schistosomiasis" or "in schistosomiasis endemic areas" should be added to the end of the sentence.*

We agree and have added the clause “in schistosomiasis endemic regions” as suggested. *Another example is the last sentence on page 18: "scenario in which schistosome-viral coinfection may be surprisingly beneficial". It would be better to have neither infection. What is surprisingly beneficial is a schistosome coinfection compared to viral infection alone.*

We agree and have revised the sentence so that it now reads, “these mouse respiratory virus data exemplify another scenario in which schistosome-viral coinfection may be surprisingly beneficial compared to viral infection alone.” *Second to last sentence in first paragraph on page 19: "...but only 6 women had confirmed infection." With what? HPV or schistosomes? Second sentence of next paragraph "...than uninfected baboons." Better to say than baboons that only had HPV infection.*

Thank you. The 6 women had confirmed schistosome infection. We have clarified both this and the sentence about baboons. Baboons with *S. mansoni* infection were compared to baboons that had neither schistosome nor HPV infection as this was a study on response to the HPV vaccine. *Third sentence in last paragraph on page 19--please indicate the timing of the praziquantel treatment that restored IgG titers. Were this titers actually "restored" in a given individual or did the praziquantel treatment group simply have a higher level of IgG than the untreated schistosome-infected group?*

We appreciate this question. The praziquantel treatment group developed a higher level of IgG than the children who were untreated at the time of immunization and were treated one week later. We have removed the word “restored” and clarified this in the text. *First line of page 23: presumably the intent is that treatment of schistosomiasis alleviates liver fibrosis in persons with hepatitis. It is no surprise that treating schistosomiasis prevents schistosomiasis-related fibrosis. Just be clear.*

We agree with the reviewer and have clarified this sentence with the clause “in schistosome-hepatitis co-infected persons.” *In discussing the human schistosome challenge model on page 23, it is important to note that these are not patent infections, thus some of the presumed benefit of the Th2 and T reg responses associated with egg deposition may not in fact be available.*

We appreciate this important point and have added it to the manuscript in the section in which we discuss the human challenge model. *Please read back through the whole manuscript and correct these and other unclear comparisons. This can be annoying but it is necessary to be very clear about which infection is being referred to in coinfection studies.*

Thank you again for these suggestions. We have carefully re-read the entire manuscript and made additional minor changes to ensure comparisons are clear and writing is straightforward. *Other small errors needing correction:*

*--the first two entries in table 1 include the journal, the rest don't*

We have modified the table so that none of the entries include the journal.

*--a quick glance shows that several references (21, 110, 130) are incomplete and other citations (112, 128) are not valid references. Please go back through and check the entire bibliography.*

Thank you for this comment. We have checked and corrected the entire bibliography.

*--in figure 2, there seems to be words missing at the end of the "Beneficial" box. Also, the increase in HIV coreceptors and macaque susceptibility is not limited to females alone. What about transmission in this figure? An older paper by Leuschter showed increased CD4 cells in semen of men with high intensity S. haematobium infection and citation 85 shows greater risk of transmitting HIV to HIV-negative partners by persons with schistosome infection.*

Thank you for these suggestions to strengthen this figure. We have fixed the missing word and modified the increase in HIV coreceptors and macaque susceptibility to not be limited to females only. We have not included transmission in the figure because the only paper that directly investigated transmission was reference 85, which was not included in the review due to its use of antibody testing. However, we have mentioned this finding about transmission in the text when we discuss reference 85, and we have also now discussed the older paper by Leutscher and another by that research group at that point in the text.

*--Table 9 first sentence on vaccine efficacy seems to have an addition or deletion word mutation (are versus). THe point about precise timing in the next block brings up an important point that probably merits a paragraph in the discussion. Age prevalence curves of the different infections may be key. For example, a person is likely to get exposed to rotavirus and childhood vaccinations prior to becoming infected with schistosomes but conversely is more likely to acquire a schistosome infection in childhood before they are exposed to sexually transmitted viruses like HPV, HCV, and HIV. Exposure to respiratory viruses may be more concurrent. Both the timing and the dose of the various infections could influence the manifestation of the coinfection.*

We have modified the first sentence in Table 9 so that it now reads, “Vaccine efficacy… in LMIC settings in which schistosomes are endemic versus LMIC settings in which schistosomes are not endemic.” We agree with your point about the importance of age prevalence curves of viral and schistosome infections. We have added a paragraph in the discussion (paragraph 6) to address the impact of timing, duration, and dose of infection.

**Reviewer 2**

*Authors should state in methods (or discussion) whether their definition of virulence (“severity of clinical disease in co-infected subjects”) coincided well with definitions used in selected/included primary manuscripts.*

We have added a sentence in the “Study Selection” section of the methods stating that our definition of virulence did indeed coincide with definitions of the studies included in the review.

*It is stated that Abruzzi et. al.” concluded that numerous studies in patients
with schistosome-HCV coinfection indicate that schistosome-hepatitis coinfections have
multiplicative, rather than additive, effects on liver disease.”
Suggest being sure that appropriate statistical approaches were used to determine “multiplicity” (or synergistic effects even if worsening”) rather than purely additive.*

Thank you for this comment. We have modified the language in this sentence to reflect the specific language used by Abruzzi and colleagues, which focuses on synergism rather than multiplicity.

*Page 5 – for reference to HCV specific intrahepatic TH1 responses, should mention these were human subjects with biopsy if that how the this was ascertained.*

We have added language to clarify that these results were biopsy-confirmed.

*In figure one would change “piece” as in “review piece” to article.*

We have changed the wording from “piece” to “article.”
